# Late-stage differentiation of embryonic pancreatic β-cells requires Jarid2

**DOI:** 10.1038/s41598-017-11691-2

**Published:** 2017-09-14

**Authors:** Sara Cervantes, Marta Fontcuberta-PiSunyer, Joan-Marc Servitja, Rebeca Fernandez-Ruiz, Ainhoa García, Lidia Sanchez, Young-Sook Lee, Ramon Gomis, Rosa Gasa

**Affiliations:** 1 Diabetes and Obesity Research Laboratory, August Pi i Sunyer Biomedical Research Institute (IDIBAPS), Rosselló 149-153, 08036 Barcelona, Spain; 2 Centro de Investigación Biomédica en Red de Diabetes y Enfermedades Metabólicas Asociadas (CIBERDEM), Barcelona, Spain; 3 University of Barcelona, Barcelona, Spain; 4 Department of Cellular and Regenerative Biology, University of Wisconsin-Madison, Madison, Wisconsin 53706 USA; 5 Department of Endocrinology and Nutrition, Hospital Clinic of Barcelona, Barcelona, Spain; 6 Department of Medicine, University of Barcelona, Barcelona, Spain

## Abstract

Jarid2 is a component of the Polycomb Repressor complex 2 (PRC2), which is responsible for genome-wide H3K27me3 deposition, in embryonic stem cells. However, Jarid2 has also been shown to exert pleiotropic PRC2-independent actions during embryogenesis. Here, we have investigated the role of Jarid2 during pancreas development. Conditional ablation of Jarid2 in pancreatic progenitors results in reduced endocrine cell area at birth due to impaired endocrine cell differentiation and reduced prenatal proliferation. Inactivation of Jarid2 in endocrine progenitors demonstrates that Jarid2 functions after endocrine specification. Furthermore, genome-wide expression analysis reveals that Jarid2 is required for the complete activation of the insulin-producing β-cell differentiation program. Jarid2-deficient pancreases exhibit impaired deposition of RNAPII-Ser5P, the initiating form of RNAPII, but no changes in H3K27me3, at the promoters of affected endocrine genes. Thus, our study identifies Jarid2 as a fine-tuner of gene expression during late stages of pancreatic endocrine cell development. These findings are relevant for generation of transplantable stem cell-derived β-cells.

## Introduction

Diabetes mellitus (DM) is a complex disease that results from failure of β-cells to secrete enough insulin to maintain normoglycemia. Seminal studies have demonstrated that it is possible to generate insulin-secreting β–cells from ESCs and iPSCs through the stepwise addition of growth factors and chemical compounds^1–3^, recapitulating the different stages of *in vivo* endocrine cell differentiation. Even though the *in vitro* generated β-cells are able to prevent or ameliorate hyperglycemia in mouse models of diabetes, their gene expression profile and functionality still differs from that of mature human β-cells^2, 3^.

The endocrine compartment of the pancreas is constituted by α- (glucagon), β- (insulin), δ- (somatostatin), PP- (pancreatic polypeptide) and ε-(ghrelin) cells, which reside in the islets of Langerhans, surrounded by exocrine tissue (acinar and ductal). Between embryonic day (e)13.5 and e15.5, the bulk of endocrine cell formation unfolds in the trunk region of the pancreatic epithelium, a process known as the secondary transition. Transient expression of the master pro-endocrine transcription factor Neurogenin3 (Ngn3) in discrete cells within this domain generates monohormonal endocrine precursors, which will activate genes necessary for their endocrine function as they become mature endocrine cell types.

Although there is a broad knowledge of the transcriptional and signaling pathways that govern pancreatic cell-fate transitions, little is known about how chromatin modifiers regulate this process^4–6^. Only in the last few years we have begun to identify the chromatin modifications that accompany gene expression changes. The Polycomb Repressive Complex 2 (PRC2) catalyzes the trimethylation of lysine 27 in the tail of Histone H3 (H3K27me3) through its enzymatic activities Ezh1 and Ezh2, resulting in transcriptional silencing. During mouse pancreas organogenesis, H3K27me3 is dynamically modified at the promoters of pancreatic and endocrine-specific genes^7, 8^. Ezh2 represses Pdx1 expression from the prospective liver domain, thus allowing liver specification while restricting the ventral pancreas^9^. Later during endocrine differentiation, Ezh2 represses endocrine cell fate thus restraining endocrine cell mass formation. Accordingly, in mouse pancreatic explants and pancreatic cells obtained from hESCs, chemical inhibition of Ezh2 resulted in increased endocrine cell differentiation^8^.

Jarid2 (jumonji, AT rich interactive domain 2) is the founding member of the Jumonji-containing family of demethylases, even though it contains aminoacid substitutions that abolish its catalytic activity, and is a facultative component of PRC2. In ESCs, Jarid2 fine-tunes H3K27me3 levels and is essential for successful ESC differentiation, most likely by priming PRC2 target genes for expression upon induction of differentiation^10, 11^. Recently, Jarid2 has been found in complexes with G9a/GLP and SETDB1 that regulate H3K9me3 levels (another repressive mark)^12–14^ and thus, it may help coordinate methylation of H3K27 and H3K9. Deletion of Jarid2 in mice results in severe abnormalities in multiple organs including brain, heart, liver, spleen and blood tissues. Jarid2 also plays important roles in skin and muscle differentiation^15–18^. Additionally, two studies aimed at identifying genes enriched during pancreatic endocrine differentiation *in vivo* in mouse embryos, reported increased expression of *Jarid2* in endocrine progenitors and descendants^19, 20^. Here we set out to determine the potential role of Jarid2 in pancreatic and endocrine cell differentiation. We show that Jarid2 is required in progenitor cells to activate the β-cell gene expression program and thus generate fully differentiated β-cells.

## Results

### Ablation of Jarid2 in pancreatic progenitors results in reduced β-cell mass

**Figure 1:**
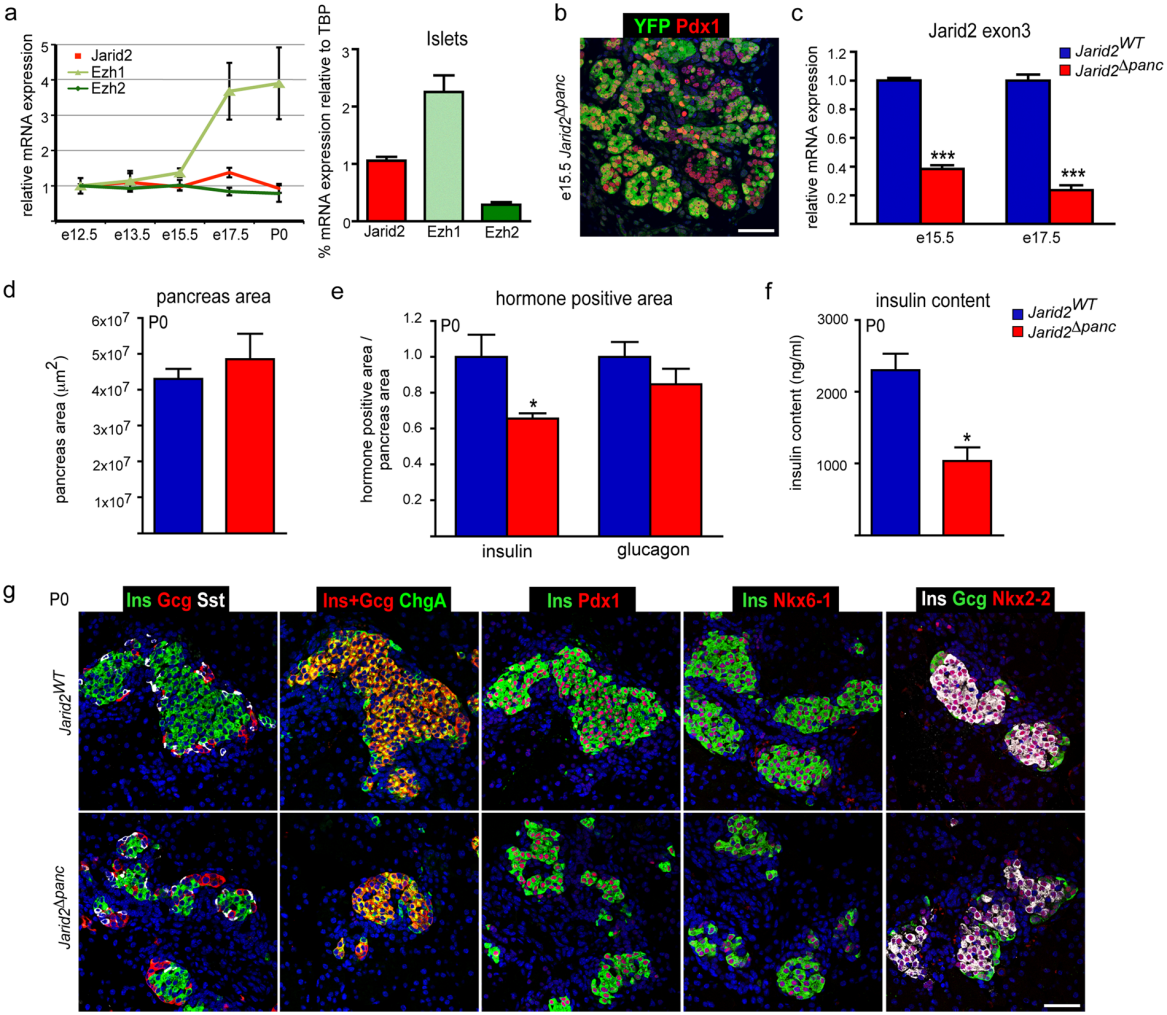
Ablation of Jarid2 in pancreatic progenitors results in reduced β-cell mass at birth. (**a**) Quantification by qRT-PCR of *Ezh1*, *Ezh2* and *Jarid2* mRNAs at the indicated embryonic stages and in islets. For the embryonic pancreases, the kinetics of expression throughout development is represented relative to the expression at e12.5, while the expression in islets is shown relative to *Tbp*. Each data point represents mean ± SEM from 2–7 independent mRNA extractions. The e12.5 and e13.5 data points are pools of several pancreases. (**b**) Immunofluorescence analysis of YFP expression (green) using an anti-GFP antibody that cross-reacts with YFP to assess recombination in *Jarid2* ^*Δpanc*^ mice at e15.5. Staining against Pdx1 (red) is used to mark the pancreatic epithelium. Nuclei were stained with Hoechst 33258 (blue). Scale bar: 50 μm. (**c**) Quantification by qRT-PCR of the relative expression of *Jarid2* mRNA at the indicated embryonic stages in *Jarid2* ^*WT*^ (n = 11 and n = 6 at e15.5 and 17.5, respectively) and *Jarid2* ^*Δpanc*^ (n = 15 and n = 5 at e15.5 and 17.5, respectively) embryonic pancreases. Primers that amplify *Jarid2* exon3 were used to detect its excision. Bars represent mean ± SEM; ***p < 0.0001. (**d**) Morphometric analysis of *Jarid2* ^*WT*^ (n = 4) and *Jarid2* ^*Δpanc*^ (n = 4) pancreatic area in newborn mice (P0). Bars represent mean ± SEM. (**e**) Morphometric analysis of *Jarid2* ^*WT*^ (n = 4) and *Jarid2* ^*Δpanc*^ (n = 4) insulin and glucagon positive area in newborn mice (P0). Bars represent mean ± SEM; *p < 0.05. (**f**) Total insulin content from *Jarid2* ^*WT*^ (n = 5) and *Jarid2* ^*Δpanc*^ (n = 3) pancreases from newborn mice (P0) was quantified by ELISA. Bars represent mean ± SEM; *p < 0.01. (**g**) Immunofluorescence stainings against hormones, endocrine cell markers, and endocrine transcription factors in *Jarid2* ^*WT*^ and *Jarid2* ^*Δpanc*^ pancreases from newborn mice (P0) using anti-insulin, anti-glucagon, anti-somatostatin, anti-chromograninA (ChgA), anti-Pdx1, anti-Nkx6-1 and anti-Nkx2-2 antibodies. Nuclei were stained with Hoechst 33258 (blue). Endocrine clusters appeared disaggregated in *Jarid2* ^*Δpanc*^ but express the appropriate markers and factors. Scale bar: 50 μm.

Quantitative RT-PCR using whole pancreas lysates showed that *Jarid2* is expressed throughout pancreatic development. While *Jarid2* mRNA levels are maintained relatively constant*, Ezh1* expression is markedly increased and *Ezh2* mRNA diminished at late gestation. In adult islets, *Jarid2* mRNA is expressed at intermediate levels between *Ezh1* and *Ezh2* (Fig. 1a).

To investigate the role of Jarid2 in pancreatic development, we generated mice in which *Jarid2* was ablated from pancreatic progenitors (*Pdx1-Cre;Jarid2* ^*flox/flox*^, from here on *Jarid2* ^*Δpanc*^). In the absence of a reliable Jarid2 antibody for immunostaining, we introduced the reporter *R26-YFP* allele to evaluate the extent of Cre-mediated recombination. Immunostaining against YFP revealed that at e15.5, most epithelial cells had recombined, except for some cells located in the trunk and tip areas, thus proving epithelium-specific but mosaic Pdx1-driven Cre recombination (Fig. 1b). To specifically determine the extent of *Jarid2* downregulation, we quantified expression of the loxP-flanked *Jarid2* exon3 and detected a 62% and 77% reduction compared to controls at e15.5 and e17.5, respectively (Fig. 1c). The remaining *Jarid2* expression is possibly due to non-recombined epithelial cells and non-epithelial cell types, such as mesenchymal cells, which are present in reduced proportion as the pancreas develops^21^. The fact that the *Jarid2* exon3 excision increased in older embryos (e17.5 vs e15.5) supports this notion.

At birth, the pancreas of *Jarid2* ^*Δpanc*^ mice appeared normal in morphology and size, as shown by quantification of pancreatic area (Fig. 1d). Yet, *Jarid2* ^*Δpanc*^ presented a 40% reduction in insulin positive fractional area and a marginal reduction in glucagon positive area (Fig. 1e). Accordingly, pancreatic insulin content was reduced by 50% (Fig. 1f). To examine the endocrine compartment in more detail, we analyzed a series of endocrine and β-cell markers in *Jarid2* ^*WT*^ and *Jarid2* ^*Δpanc*^ tissues. At P0, endocrine cell clusters formed, although they appeared less compact and disaggregated in mutants than controls. These clusters in *Jarid2* ^*Δpanc*^ tissues appropriately expressed key transcription factors such as Pdx1, Nkx6-1, Nkx2-2, and the pan-endocrine marker and secretory granule protein ChromograninA or ChgA (Fig. 1g). The reduction in β-cell fractional area did not alter serum glucose levels in newborn animals (not shown). Adult *Jarid2* ^*Δpanc*^ mice also displayed normal fasting blood glucose, but were glucose intolerant at 9–11wks, while showing unaltered body weight (Supplementary Fig. S1). β-cell mass and islet size distribution were comparable to that of controls (Supplementary Fig. S1), indicating that postnatal β-cell expansion is able to compensate the reduction in β-cell number observed at birth. Yet, despite reaching normal β-cell mass, adult *Jarid2* ^*Δpanc*^ mice are glucose intolerant, which suggests that generated β-cells are not fully functional. Whether this is consequence of aberrant β-cell formation due to *Jarid2* loss during pancreatic development or it results from the compensatory postnatal growth remains to be determined.

### Endocrine cell formation is perturbed during the secondary transition in *Jarid2*^*Δpanc*^ mice

**Figure 2:**
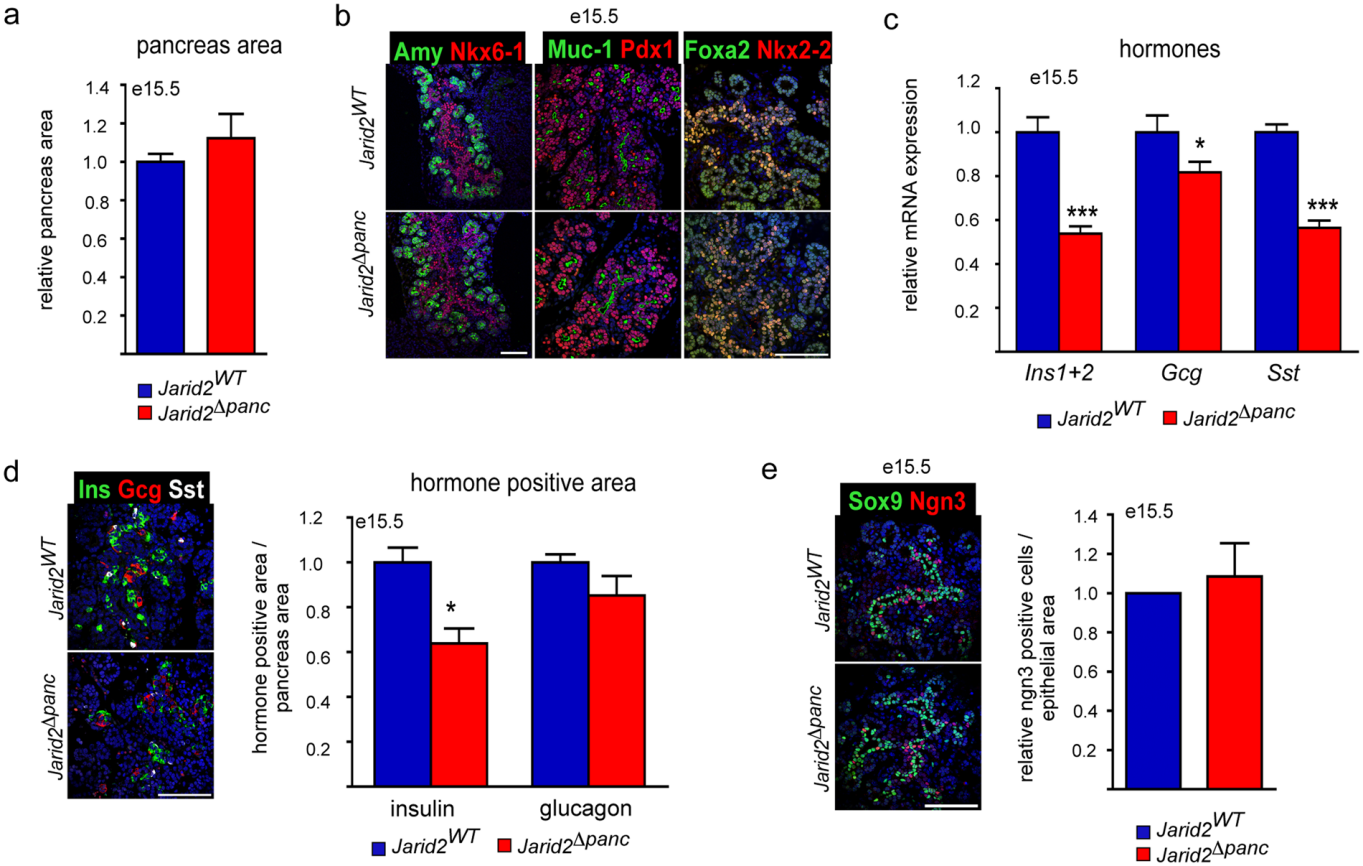
Ablation of Jarid2 in pancreatic progenitors results in reduced endocrine cell formation during the secondary transition. (**a**) Morphometric analysis of *Jarid2* ^*WT*^ (n = 3) and *Jarid2* ^*Δpanc*^ (n = 3) pancreatic area at e15.5. Bars represent mean ± SEM. (**b**) Immunofluorescence stainings against the indicated general (Pdx1, Foxa2), exocrine (Amy), ductal (Muc-1) and endocrine (Nkx6-1, Nkx2-2) cell markers in *Jarid2* ^*WT*^ and *Jarid2* ^*Δpanc*^ e15.5 pancreases confirms that the corresponding pancreatic compartments are appropriately formed at this stage. Nuclei were stained with Hoechst 33258 (blue). Scale bar: 100 μm. (**c**) Quantification by qRT-PCR of the relative expression of the three main pancreatic hormones *Ins1*+*2, Gcg* and *Sst* in *Jarid2* ^*WT*^ (n = 11) and *Jarid2* ^*Δpanc*^ (n = 15) e15.5 pancreases. Bars represent mean ± SEM; *p < 0.05, ***p < 0.0001. (**d**) Morphometric analysis of *Jarid2* ^*WT*^ (n = 3) and *Jarid2* ^*Δpanc*^ (n = 3) insulin and glucagon positive area in e15.5 embryos. Columns represent mean ± SEM *p < 0.05. Representative image of the immunofluorescence staining against the three main pancreatic hormones, insulin (green), glucagon (red) and somatostatin (white) is shown on the right. Nuclei were stained with Hoechst 33258 (blue). Scale bar: 100 μm. (**e**) Immunofluorescence stainings against Sox9 (green) and Ngn3 (red) in *Jarid2* ^*WT*^ and *Jarid2* ^*Δpanc*^ shows that these markers are appropriately expressed at this stage. Nuclei were stained with Hoechst 33258 (blue). Scale bar: 100 μm. On the right, morphometric analysis of the number of Ngn3+ cells per epithelial (Pdx1+) area. Columns represent mean ± SEM (n = 4–5) and values are expressed relative to controls, which are given the number of 1.

As the bulk of β-cell neogenesis occurs during the secondary transition, we studied e15.5 pancreata from *Jarid2* ^*Δpanc*^ embryos and control littermates. Pancreatic morphology and size appeared normal, which was confirmed by quantification of pancreatic area on tissue sections (Fig. 2a). Likewise, endocrine (Pdx1, Nkx6-1, Nkx2-2), exocrine (Amy), ductal (Muc-1), and bipotent endocrine/ductal cell markers (Nkx6-1, Nkx2-2) were present and normally distributed (Fig. 2b). To uncover potential defects in the endocrine compartment, we quantified the mRNA encoding the three main pancreatic hormones, insulin (*Ins1* and *Ins2* genes), glucagon and somatostatin, in whole pancreatic lysates. *Insulin* and *somatostatin* expression were reduced by 50% while *glucagon* was reduced by only 18% (Fig. 2c). We next performed morphometric quantification of the endocrine positive area to determine whether the observed changes in expression correlated with reduced cell numbers. We found a 40% reduction in insulin positive area in *Jarid2* ^*Δpanc*^ embryos, while that of glucagon was not significantly affected (Fig. 2d). Immunofluorescence staining against Sox9+ bipotent ductal/endocrine demonstrated the normal distribution of this cell population (Fig. 2e). Likewise, distribution and number of Ngn3+ endocrine progenitors was not affected by Jarid2 loss (Fig. 2e). Altogether, these results reveal that Jarid2 is dispensable for endocrine specification but it is required for differentiation of β- and δ-cells during the secondary transition.

### Jarid2 is necessary for the transcriptional activation of pancreas-specific genes

**Figure 3:**
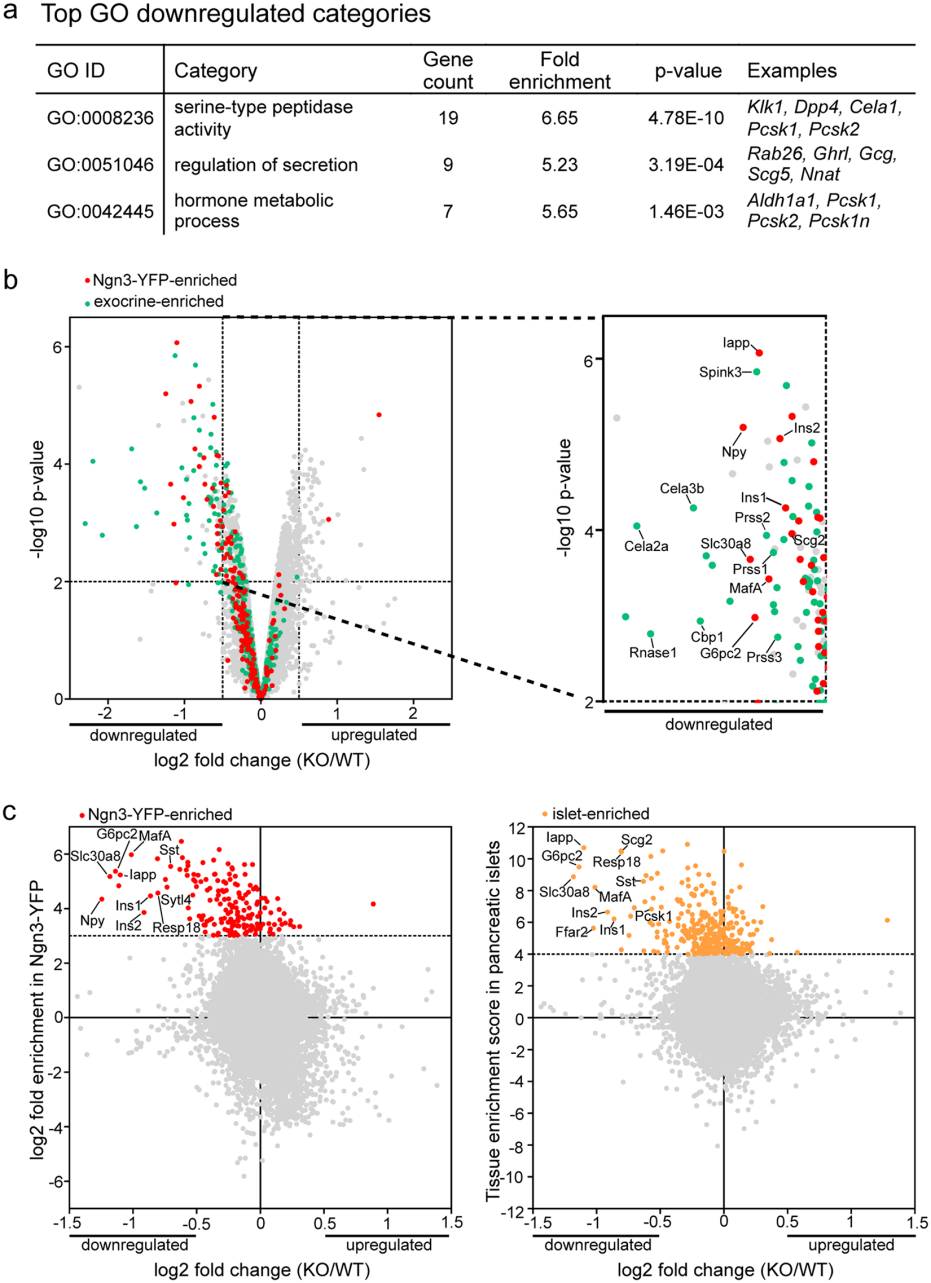
Jarid2 is necessary for the transcriptional activation of pancreas-specific genes. (**a**) Top GO categories identified by Gene Ontology analysis of the genes downregulated in the absence of Jarid2 in e15.5 mouse embryonic pancreases. (**b**) Volcano plot of all the genes represented in the microarray relating changes of gene expression and the corresponding statistical significances in *Jarid2* ^*WT*^ vs *Jarid2* ^*Δpanc*^ pancreases (X axis). Genes enriched in the Ngn3-YFP^+^ and exocrine cell populations are marked in red and green, respectively. Note that most of the genes in these two categories fall towards the left side of the plot, indicating that differentially expressed pancreas-specific genes are mainly downregulated in the absence of Jarid2. The identity of some of the most downregulated exocrine genes is indicated on the magnified graph. (**c**) Scatter plot of all genes represented in the microarray correlating their changes in gene expression in *Jarid2* ^*WT*^ vs *Jarid2* ^*Δpanc*^ pancreases (X axis) with their enrichment in Ngn3-YFP+ cells (left) and pancreatic islets^40^ (Y axis). Genes enriched in the Ngn3-YFP^+^ and islet cell populations are marked in red and orange on the left and right panels, respectively. Note that many of the genes most downregulated in *Jarid2* ^*Δpanc*^ vs *Jarid2* ^*WT*^ pancreases are enriched in Ngn3-YFP+ and/or islet cells. The identity of some of these genes is indicated on graphs.

To gain insights into the mechanisms underlying these differentiation defects, we performed a microarray analysis using RNA from whole *Jarid2* ^*Δpanc*^ and control e15.5 pancreases. Among the 217 genes that were significantly downregulated, Gene Ontology clustering revealed that the three top GO categories corresponded to a signature of pancreatic functions (peptidase activity, regulation of secretion and hormone metabolic process) (Fig. 3a). We next interrogated how exocrine-enriched and endocrine-precursor-enriched genes were affected in the absence of Jarid2. A gene set of exocrine-enriched genes was generated from a previous study comparing the gene expression profiles of a panel of mouse tissues that included the pancreatic exocrine compartment as detailed in Methods^22^. On the other hand, genes enriched in Ngn3-positive endocrine progenitors were identified from a gene expression profiling dataset obtained from YFP-positive cells in the e15.5 embryonic pancreases of mice harboring a YFP transgene inserted at the 3′UTR of *Ngn3* (*Ngn3* ^*eYFP*/+^ knock-add-on mice)^19, 23^. Remarkably, the top-most downregulated genes in *Jarid2* ^*Δpanc*^ pancreases were enriched in genes selectively expressed in endocrine and exocrine cells (red and green dots in the top-left quadrant of Fig. 3b, respectively), thus revealing a prominent role for Jarid2 in the regulation of pancreas-specific genes. Among the exocrine-enriched genes downregulated, we found several genes encoding pancreatic enzymes, including elastases (*Cela2a*, *Cela3b*, *Cela1*), *Cbp1*, Serine proteases (*Prss1*, *Prss2*, *Prss3*) and *Amy1*. Among the endocrine-enriched genes downregulated, we found *MafA*, *Ins1*, *Ins2*, *Iapp*, *G6pc2*, *Npy*, *Scg2* and *Slc30a8*. These results underscore a role for Jarid2 in the activation of both the exocrine and endocrine gene expression programs. However, the fact that pancreas size and morphology appeared normal at birth (Fig. 1d), the unaltered body weight of adult *Jarid2* ^*Δpanc*^ mice (Fig. S1), and the apparently unaltered amylase expression pattern at e15.5 (Fig. 2b), indicated that the expression changes detected in exocrine genes did not result in physiological defects in this compartment.

In contrast, the altered phenotype of the embryonic endocrine compartment resulted in reduced β-cell mass and alterations in glucose homeostasis, defects that prompted us to perform a more detailed inspection of endocrine-precursor-enriched and islet-enriched genes. A gene set of islet-enriched genes was generated comparing the gene expression profiles of a panel of mouse tissues including mouse pancreatic islets^22^. We found that the majority of genes enriched in endocrine progenitors (Ngn3-YFP-enriched) and islets (red and yellow dots, respectively) remained unchanged (Fig. 3c). However, the genes with the highest enrichment scores in both endocrine progenitors (Ngn3-YFP) and islets (i.e. *MafA*, *Ins1*, *Iapp*, *G6pc2* and *Slc30a8*) were downregulated in *Jarid2* ^*Δpanc*^ samples, indicating that loss of Jarid2 disrupts the expression of a subset of endocrine genes. Remarkably, the expression of genes known to be required for initial endocrine specification and/or differentiation such as *Ngn3*, *Pax4*, *Rfx6*, *Arx*, *Myt1*, *Nkx2-2* and *Isl1*, remained unchanged in the absence of Jarid2 (Table S1).

**Figure 4:**
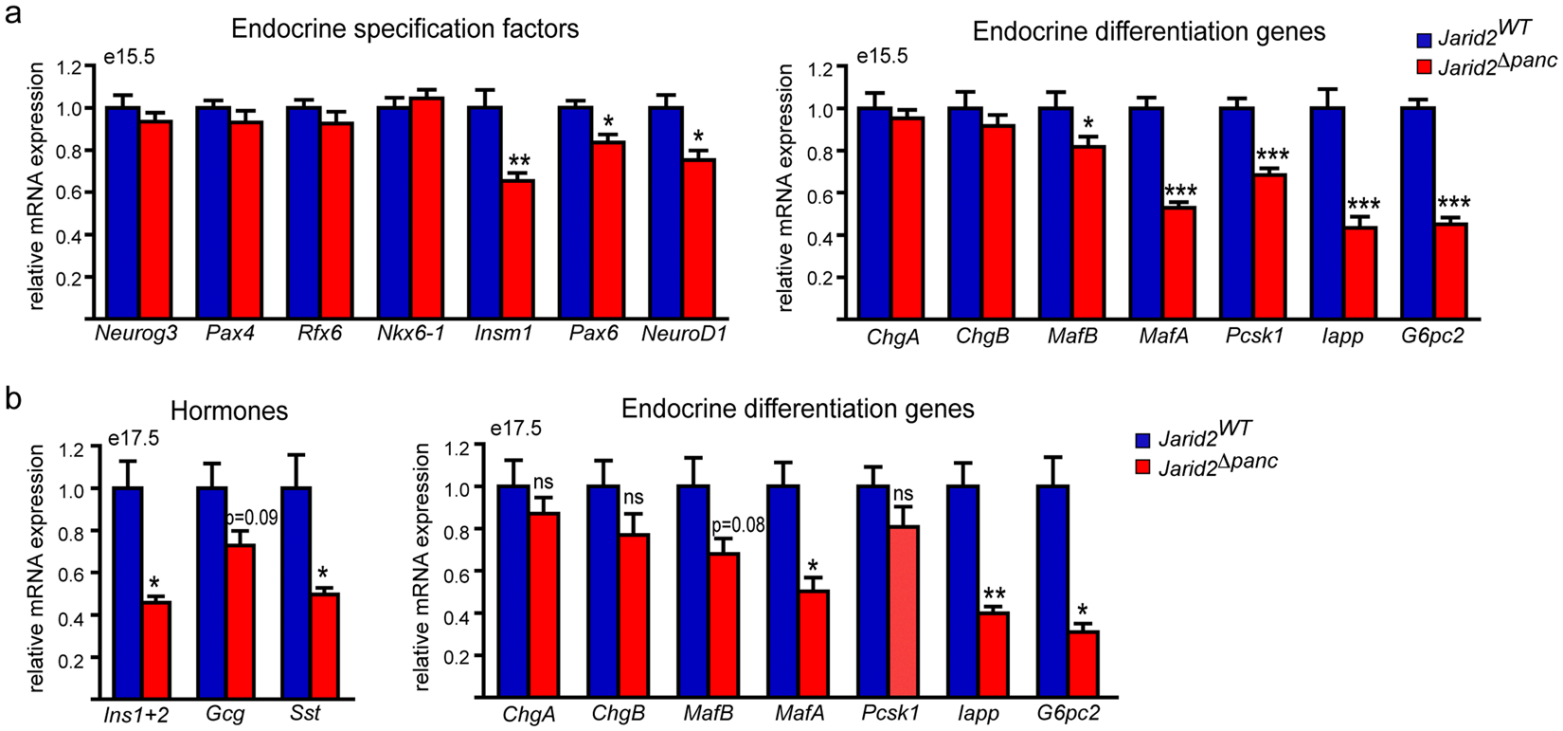
Jarid2 is required to complete endocrine cell differentiation. (**a**) Quantification by qRT-PCR of the relative expression (mRNA) of the indicated endocrine specification factors (left) and differentiation genes (right) in *Jarid2* ^*WT*^ (n = 11) and *Jarid2* ^*Δpanc*^ (n = 15) e15.5 pancreases. Bars represent represent mean ± SEM; *p < 0.05, **p < 0.001, ***p < 0.0001. (**b**) Quantification by qRT-PCR of the relative expression of the three main pancreatic hormones (left) and the indicated endocrine differentiation genes (right) in *Jarid2* ^*WT*^ (n = 5) and *Jarid2* ^*Δpanc*^ (n = 6) e17.5 pancreases. Bars represent mean ± SEM; *p < 0.05, **p < 0.001.

We validated these observations by qRT-PCR in a larger set of samples and confirmed that, only a small fraction of the early endocrine specification genes appeared modestly reduced by loss of Jarid2 (i.e. *Pax6*, *Insm1* and *NeuroD1*) (Fig. 4a). In contrast, expression of several key β-cell differentiation/maturation genes (*MafB*, *MafA*, *Pcsk1*, *Iapp* and *G6pc2*), in addition to the hormones (Fig. 2c), was reduced (Fig. 4a). Intriguingly, expression of *ChgA* and *ChgB*, early markers of committed embryonic endocrine cells which are still detectable in de-differentiated insulin-negative β-cells^24^, was not altered in *Jarid2* ^*Δpanc*^ (Fig. 4a). These latter results were validated by morphometric analysis of ChgA+ cells (Supplementary Fig. S2). Further, the relative proportion of double positive ChgA+/hormone+ was not significantly different between mutants and controls (Supplementary Fig. S2). Hence, loss of Jarid2 does not appear to impact commitment of the endocrine cell population.

Notably, analysis of gene expression in e17.5 embryos revealed that the defects in the expression of the three main pancreatic hormones as well as β-cell differentiation/maturation genes persist at later stages (Fig. 4b). The fact that most of the endocrine genes downregulated in *Jarid2* ^*Δpanc*^ embryos participate in β-cell maturation and/or function rather than initial endocrine cell specification and commitment, suggests that islet endocrine cells form but do not fully differentiate in the absence of Jarid2.

### Jarid2 is required downstream of Ngn3

**Figure 5:**
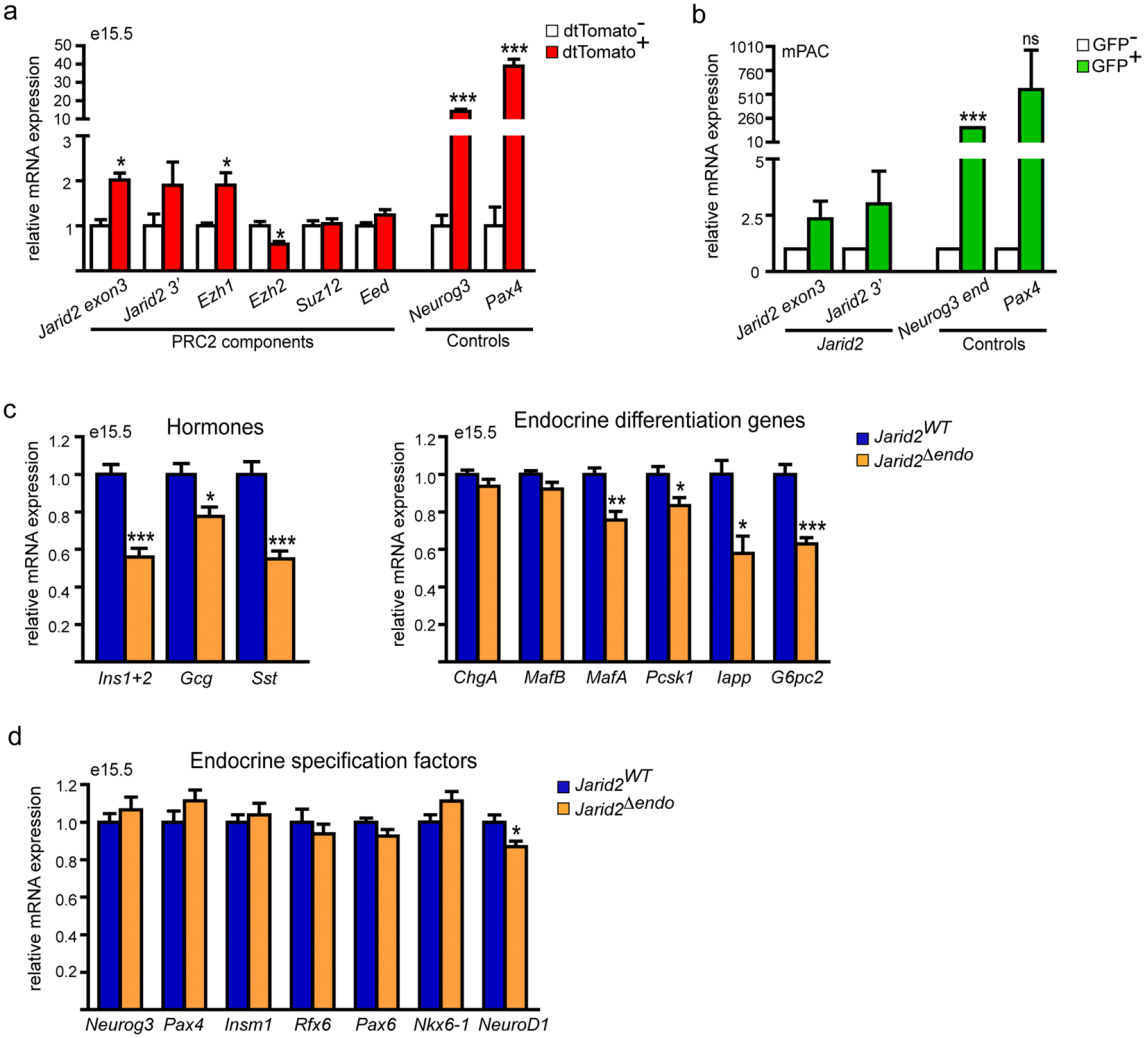
Jarid2 is required downstream of Ngn3 to complete endocrine cell differentiation. (**a**) Quantification by qRT-PCR of the relative expression of the indicated PRC2 components in *Ngn3-Cre;dtTomato* negative and positive cells from e15.5 pancreases. *Jarid2* and *Ezh1* are significantly enriched in endocrine cells while *Ezh2* is less abundant. *Neurog3* and *Pax4* expression are shown as controls. Bars represent mean ± SEM of 3–5 independent isolations; *p < 0.05, ***p < 0.0001. (**b**) Quantification by qRT-PCR of the relative expression of *Jarid2* in Ad:Ngn3-GFP transduced (GFP+) vs non-transduced (GFP-) mPAC cells. Note that *Jarid2* is modestly upregulated by Neurog3 expression. Expression of *Neurog3* (endogenous) and *Pax4* genes are shown as controls. Bars represent mean ± SEM of 3 independent experiments; ***p < 0.0001. (**c**) Quantification by qRT-PCR of the relative expression of the three main pancreatic hormones (left) and the indicated endocrine differentiation genes (right) in *Jarid2* ^*WT*^ (n = 11) and *Jarid2* ^*Δendo*^ (n = 12) e15.5 pancreases. Bars represent mean ± SEM; *p < 0.05, **p < 0.001, ***p < 0.0001. (**d**) Quantification by qRT-PCR of the relative expression of the indicated endocrine differentiation factors in *Jarid2* ^wt^ (n = 11) and *Jarid2* ^*Δendo*^ (n = 12) e15.5 pancreases. Bars represent mean ± SEM *p < 0.05. Note that except for *NeuroD1*, key endocrine specification factors remained unchanged.

Our results indicate that Jarid2 function is required most likely after Ngn3, since expression of early endocrine specification genes including *Neurog3* remained unaltered. As an initial step, we assessed whether Jarid2 abundance was associated with endocrine differentiation. We isolated endocrine progenitors and their descendants from *Ngn3-Cre;tdTomato* e15.5 embryonic pancreases by FACS sorting and quantified expression of *Jarid2* and of several PRC2 components. Interestingly, we found that while *Ezh2* expression was reduced in the endocrine compartment, *Ezh1* and *Jarid2* RNAs were enriched by two-fold (Fig. 5a), indicating that *Jarid2* expression is increased in endocrine cells. To test this possibility, we used an adenovirus encoding a GFP-tagged version of Ngn3 (Ad.Ngn3:GFP) to transduce mPAC cells and, in agreement with our finding *in vivo*, *Jarid2* expression was augmented in transduced (GFP+) cells (Fig. 5b). Altogether, our results show that expression of PRC2 components and, specifically, expression of Jarid2, is regulated in the endocrine compartment.

To experimentally test a cell-autonomous requirement for Jarid2 in the endocrine lineage, we ablated Jarid2 in endocrine progenitors (*Ngn3-Cre;Jarid2* ^*flox/flox*^, from here on *Jarid2* ^*Δendo*^). We obtained embryonic pancreas at e15.5, which appeared normal in size and morphology (not shown). As in *Jarid2* ^*Δpanc*^, we found a 50% reduction in the expression levels of *insulin* and *somatostatin*, while *glucagon* gene expression exhibited a lower decrease (Fig. 5c). Of all the early specification genes analyzed, only *NeuroD1* appeared downregulated, albeit modestly (Fig. 5d), whereas the expression of several genes involved in β-cell differentiation and function such as *MafA, Pcsk1*, *Iapp*, and *G6pc2* were diminished (Fig. 5c).

We also asked whether Jarid2 was required in differentiating β-cells. For this purpose, we generated mice in which *Jarid2* was ablated from β-cells at the onset of *insulin* gene expression (*Rip-Cre;Jarid2* ^*flox/flox*^, from here on *Jarid2* ^*Δbeta*^). We performed a morphometric analysis of both insulin and glucagon cell areas, and found that they remained unaltered in *Jarid2* ^*Δbeta*^ newborns (Supplementary Fig. S3). These results show that Jarid2 depletion in embryonic β-cells has no significant impact on β-cell numbers at birth.

Overall, these findings support that Jarid2 major role in β-cell mass establishment is downstream of Ngn3 and before or shortly after the *insulin* gene is expressed.

### Reduced β-cell proliferation during late gestation in *Jarid2*^*Δpanc*^ mice

**Figure 6:**
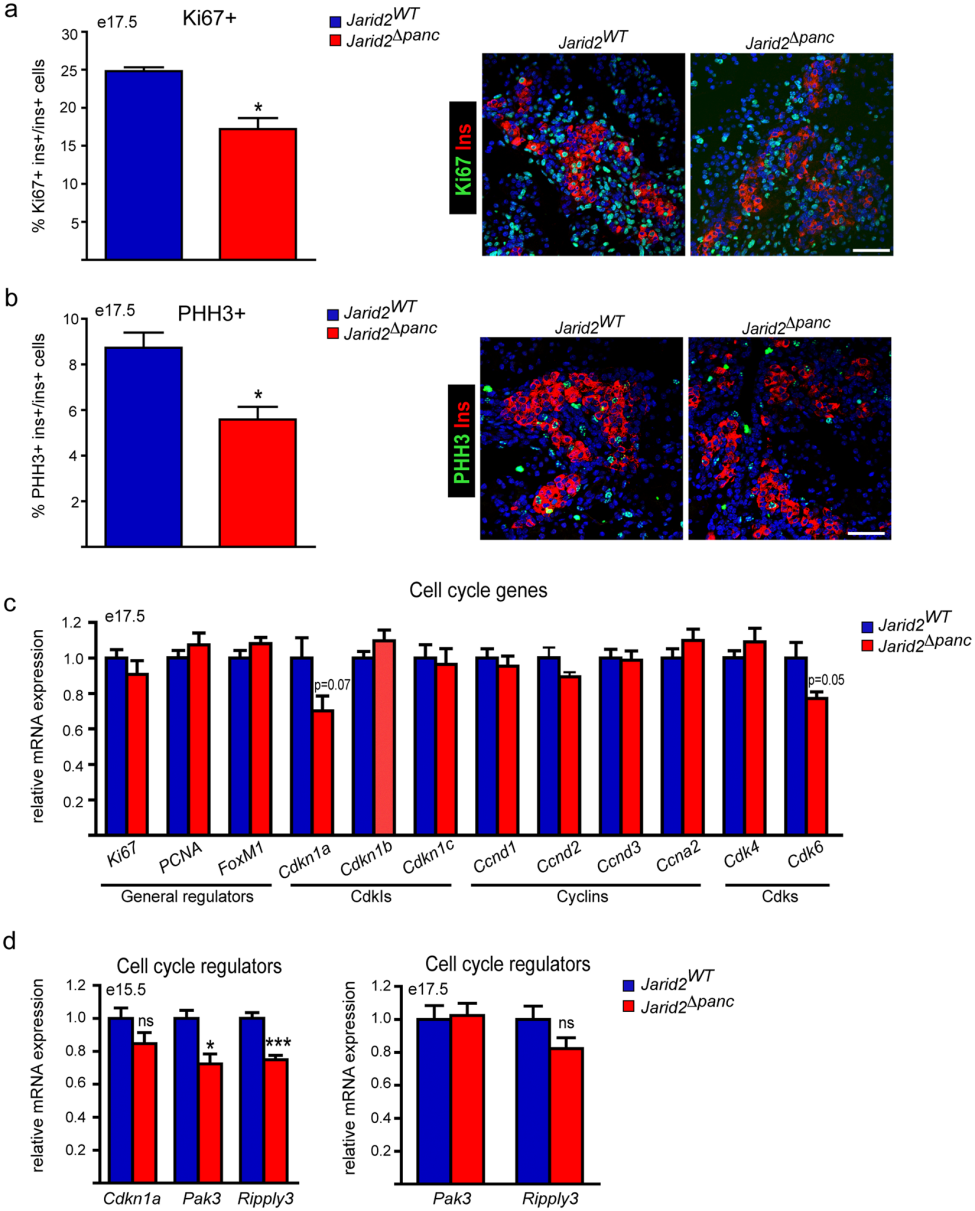
Ablation of Jarid2 in pancreatic progenitors results in reduced prenatal β-cell proliferation. (**a**) Percentage of β-cells (ins^+^) that are Ki67^+^ in *Jarid2* ^*WT*^ (n = 3) and *Jarid2* ^*Δpanc*^ (n = 3) pancreases from e17.5 embryos. Bars represent mean ± SEM; *p < 0.05. A representative image of the insulin (red) and Ki67 (green) immunofluorescence staining is shown on the right. Scale bar: 50 μm. (**b**) Percentage of β-cells (ins^+^) that are PHH3^+^ in *Jarid2* ^*WT*^ (n = 4) and *Jarid2* ^*Δpanc*^ (n = 3) pancreases from e17.5 embryos. Bars represent mean ± SEM; *p < 0.05. A representative image of the insulin (red) and PHH3 (green) immunofluorescence staining is shown on the right. Scale bar: 50 μm. (**c**) Relative expression of the indicated cell cycle regulators in *Jarid2* ^*WT*^ (n = 6) and *Jarid2* ^*Δpanc*^ (n = 5) e17.5 pancreases. Bars represent mean ± SEM. Note that most of the genes assayed remained unchanged. (**d**) Quantification by qRT-PCR of the relative expression of the cell cycle regulators *Cdkn1a*, *Pak3* and *Ripply3* at the indicated embryonic stages in *Jarid2* ^*WT*^ (n = 11 and n = 6 at e15.5 and 17.5, respectively) and *Jarid2* ^*Δpanc*^ (n = 15 and n = 5 at e15.5 and 17.5, respectively) embryonic pancreases. Bars represent mean ± SEM. *p < 0.05, ***p < 0.0001.

Jarid2 has been shown to inhibit cell proliferation during cardiogenesis and neurogenesis^14, 25–28^, and is required for the scheduled reactivation of epidermal progenitor and stem cells^16^. Thus, we asked whether de-regulated proliferation in embryonic β-cells^29, 30^ might also contribute to the reduction in β-cell mass in *Jarid2* ^*Δpanc*^ mice. We quantified cycling insulin positive cells at e17.5 by immunostaining using an anti-ki67 antibody and found a ∼30% decrease in *Jarid2* ^*Δpanc*^ relative to controls (Fig. 6a). We also immunostained for Phospho-HistoneH3 (PHH3) and observed a ∼35% reduction in the proportion of β-cells that are actively dividing (late G2 and M phases) in *Jarid2* ^*Δpanc*^ pancreases (Fig. 6b).

We next investigated the expression of cell cycle genes in e17.5 whole pancreas. We found that among the general cycle regulators analyzed, only *Cdkn1a* (p21) and *Cdk6* appeared modestly downregulated (Fig. 6c). We also assessed by manual inspection the behavior of general cell cycle regulators in our microarray data obtained at e15.5. Expression of the general cell cycle regulators did not change in the absence of Jarid2, except for *Cdkn1a*, which appeared downregulated, although not significantly when we validated these results by qRT-PCR using a larger set of samples (Fig. 6c). Interestingly, two less known cell cycle regulators, *Pak3* and *Ripply3*, which have been recently shown to limit endocrine cell proliferation^31, 32^, appeared downregulated. We validated these observations by qRT-PCR and found that both *Pak3* and *Ripply3* were decreased by ∼25% at e15.5 (Fig. 6c), but these changes did not persist later at e17.5 when β-cells undergo replication (Fig. 6d).

In addition, we determined whether apoptosis could also be affecting the resulting β-cell mass. Tunel assays detected no apoptotic β-cells in both control and *Jarid2* ^*Δpanc*^ pancreases at e17.5 (data not shown). Altogether, these results demonstrate that loss of Jarid2 impairs proliferation but not survival of embryonic β-cells. Because β-cell formation appears not to be affected in *Jarid2* ^*Δbeta*^, it is plausible that the defects in proliferation are determined at precursor stages.

### Jarid2 as a regulator of H3K27me3 and RNAPII-Ser5P during pancreatic endocrine cell differentiation

Jarid2 interacts with PRC2, and G9a/GLP and SETDB1, which bear specificity towards H3K27 and H3K9, respectively. To explore the molecular function of Jarid2 in the context of pancreas development, we first quantified H3K27me3 and H3K9me2 in total pancreatic lysates from individual e15.5 embryonic pancreas by western blot. We found no differences between control and *Jarid2* ^*Δpanc*^, although H3K27me3 appeared marginally increased (Supplementary Fig. S4). This lack of broad defects was not due to compensatory changes in PRC2 components expression in the absence of Jarid2 (Supplementary Fig. S4).

**Figure 7:**
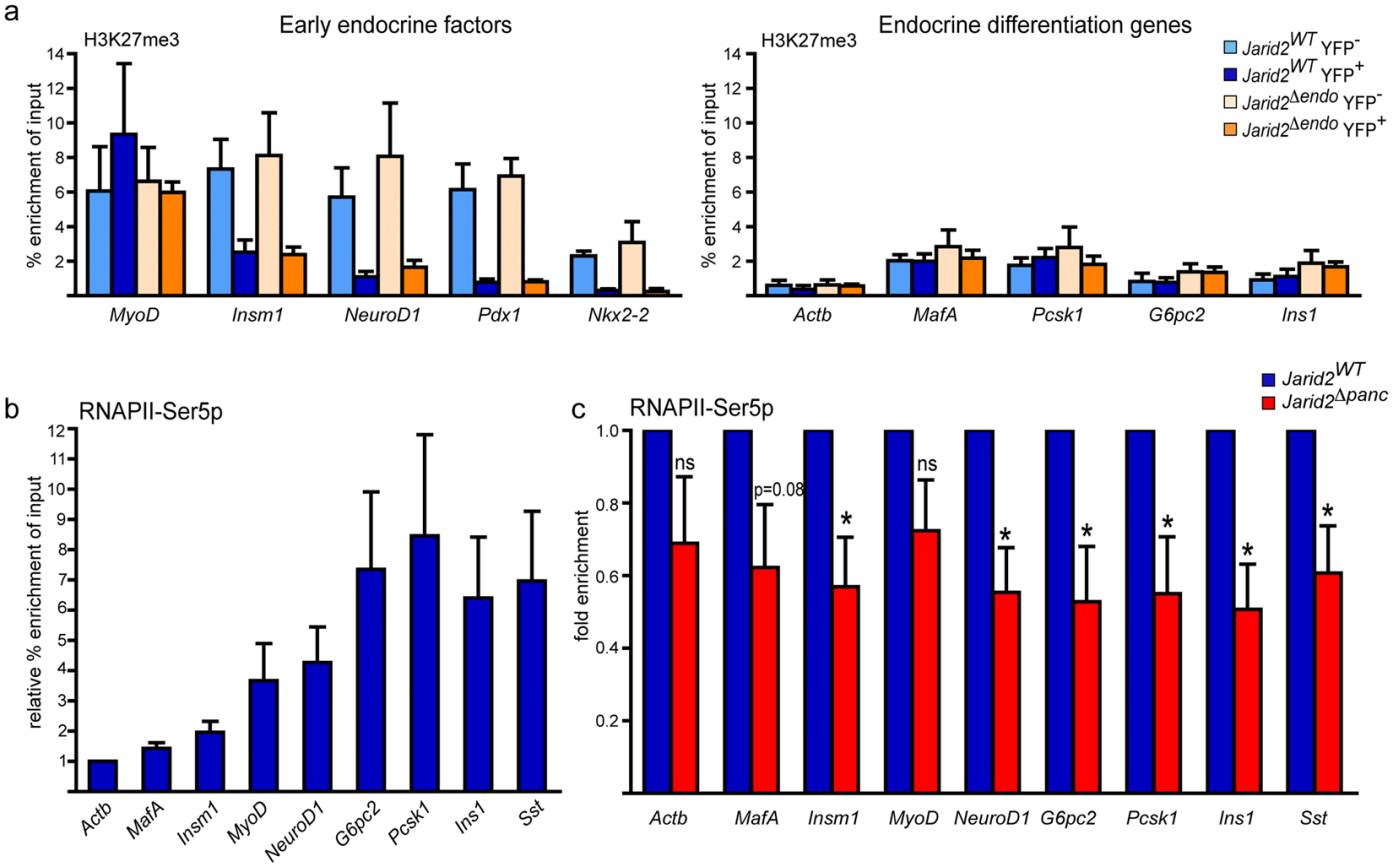
Jarid2 modulates the deposition of RNAPII-Ser5p at the promoters of endocrine genes. (**a**) Chromatin immunoprecipitation assays to determine H3K27me3 abundance at the promoter regions of early endocrine transcription factors and endocrine differentiation genes in the indicated cell populations isolated from *Ngn3-Cre;Jarid2* ^*flox/flox*^ *;R26-YFP* (*Jarid2* ^*Δendo*^) and *Ngn3-Cre;Jarid2* ^+/+^ *;R26-YFP* (*Jarid2* ^*WT*^) pancreases at e15.5. Data is presented as % enrichment of input. *MyoD* and *Actb* are shown as positive or highly enriched and negative controls, respectively. Bars represent mean ± SEM of 4 independent experiments. (**b**) Chromatin immunoprecipitation assays to determine RNAPII-Ser5p abundance at the promoter of the indicated genes in control e15.5 pancreases. Numbers are calculated as % enrichment of input and expressed relative to *Actb*, which is given arbitrarily the value of 1. Bars represent mean ± SEM for 6 independent experiments. Note that RNAPII-Ser5p abundance varies among genes, being the highest in endocrine gene markers. (**c**) Chromatin immunoprecipitation assays to determine RNAPII-Ser5p abundance at the promoter of the indicated genes in *Jarid2* ^*WT*^ and *Jarid2* ^*Δpanc*^ e15.5 pancreases. Data is presented as fold change in % enrichment of input for 6 independent experiments. Bars represent mean ± SEM; *p < 0.05.

To investigate changes in H3K27me3 specifically in endocrine cell populations (progenitors and descendants) vs non-endocrine cell types residing in the pancreas, we isolated YFP^+^ (endocrine) and YFP^−^ (non-endocrine) cells from *Ngn3-Cre;Jarid2* ^*flox/flox*^ *;R26-YFP* (*Jarid2* ^*Δendo*^) and *Ngn3-Cre;Jarid2* ^+/+^ *;R26-YFP* (*Jarid2* ^*WT*^) pancreases at e15.5 and compared the enrichment in H3K27me3 at the promoters of both early endocrine factors and differentiation genes. Interestingly, loss of Jarid2 did not change the enrichment in H3K27me3 in any of the genes tested. The enrichment in H3K27me3 at the promoters of the early endocrine factors *Insm1*, *NeuroD1, Pdx1 and Nkx2-2* in non-endocrine cells (YFP^−^) was high and diminished in endocrine progenitors and descendants (YFP^+^) both in the presence and absence of Jarid2 (Fig. 7a). Conversely, the enrichment in H3K27me3 at the promoters of the endocrine differentiation genes *MafA*, *Pcsk1*, *G6pc2* and *Ins1* was lower in non-endocrine cells (YFP^−^) as compared to that of the early endocrine factors and did not decrease further in endocrine progenitors and descendants (YFP^+^) (Fig. 7a). Again, *Jarid2* ablation had no impact in this pattern. Altogether, these experiments suggest that the defects in the initial expression of endocrine differentiation genes observed in *Jarid2* ^*Δpanc*^ and *Jarid2* ^*Δendo*^ are likely independent of a potential role of *Jarid2* in the regulation of H3K27me3.

Jarid2 is involved in the correct deposition of RNAP phosphorylated at Ser5 (RNAPII-Ser5P) at PRC2 target gene promoters in stem cells^33^. This post-translational modification is important for transcriptional priming of genes^34^. Therefore, we hypothesized that, similar to stem cells, Jarid2 might be regulating deposition of RNAPII-Ser5P at endocrine gene promoters. Thus, we analyzed RNAPII-Ser5P enrichment by chromatin immunoprecipitation in *Jarid2* ^*WT*^ and *Jarid2* ^*Δpanc*^ e15.5 pancreases. Given the fact that efficient ChIP using a phospho-specific antibody requires a high amount of starting material, we used whole pancreatic cell lysates. Analysis of the enrichment of RNAPII-Ser5P revealed that RNAPII-Ser5P levels varied greatly among the promoters of the genes studied, being particularly high at promoters of the endocrine genes *NeuroD1*, *G6pc2*, *Pcsk1*, *Ins1* and *Sst* (Fig. 7b). Remarkably, RNAPII-Ser5P levels were significantly reduced by ∼40% in *Jarid2* ^*Δpanc*^ pancreases compared to controls (Fig. 7c). Altogether, our results suggest that Jarid2 plays a role in priming endocrine genes for their appropriate expression during endocrine differentiation.

## Discussion

Results presented in this study demonstrate that Jarid2 is required during mouse pancreatic endocrine cell formation to establish the proper β-cell population at birth. Thus, ablation of Jarid2 in pancreatic and endocrine progenitors disrupts activation of the β-cell gene expression program as well as prenatal β-cell expansion leading to reduced β-cell mass in newborn mice.

In the absence of Jarid2, fewer insulin-expressing β-cells form during the secondary transition. Our genome-wide expression analysis reveals that loss of Jarid2 does not broadly affect pancreatic endocrine gene expression. Instead, only a small subset of endocrine genes is significantly reduced upon Jarid2 loss. Several of these genes code for functional and mature β-cell markers, while genes involved in early endocrine specification remain unaffected. Interestingly, Jarid2 expression is enriched, in endocrine progenitors and descendants *in vivo* and induced upon overexpression of Ngn3 in mPAC cells, indicating that *Jarid2* itself may constitute part of the endocrine differentiation program.

Our experiments comparing isolated endocrine and non-endocrine cells rule out a major role of Jarid2 in the regulation of H3K27me3 levels downstream of Ngn3. Of all the 217 genes downregulated in the absence of Jarid2, it is possible that neither endocrine transcription factors nor differentiation genes are direct targets, which might explain why we did not detect changes in the enrichment in H3K27me3 at their promoters. However, it is also possible that chromatin immunoprecipitation assays are not powerful enough to detect subtle changes, as those caused by Jarid2 ablation. Indeed, the effects of Jarid2 loss in gene expression in the pancreas are not dramatic and Jarid2 effects on H3K27me3 in other contexts have been shown to be modest^10, 11, 35^. Nonetheless, our observation that expression of early endocrine specification transcription factors is associated with loss of H3K27me3 at their promoters, while initial embryonic expression of endocrine differentiation genes is not accompanied by loss of this mark, which is already low at these promoters (Fig. 7), raises the possibility that Jarid2 plays a bigger role in the activation of genes exhibiting low H3K27me3 enrichment.

H3K27me3 is often found together with the initiating form of RNAPII, RNAPII-Ser5P, which has been proposed to prime genes for fast and efficient activation in response to differentiation^33, 34^. Our analysis of RNAPII-Ser5P at the promoters of β-cell differentiation genes indicates that loss of Jarid2 results in diminished RNAPII-Ser5P, an alteration that may possibly account for their defective expression, as previously reported in mESCs^33^. Interestingly, our findings are in sharp contrast with the consequences of loss of Ezh2 in pancreatic progenitors. Loss of Ezh2 enhances endocrine cell induction^8^ whereas loss of Jarid2 is detrimental for the complete induction of the β-cell differentiation program, resulting in opposite phenotypes. The antagonistic outcomes of the ablation of Ezh2 and Jarid2 in the same cell populations further argue that Jarid2 would function as a transcriptional activator rather than a repressor.

Among the genes whose expression is reduced in *Jarid2* ^*Δpanc*^ at e15.5 we found the cell cycle regulator *Pak3*. Interestingly, global loss of Pak3 results in increased Ngn3-positive cell numbers and a transient impairment in β-cell differentiation^32^. In the context of our study, these observations raise the possibility that *Jarid2* ^*Δpanc*^ primary defect is de-regulated cell cycle exit at e15.5, which subsequently impacts differentiation. However, endocrine cell numbers in *Pak3* ^−/−^ mice recover prenatally^32^ and mRNA levels of main components of the cell cycle machinery are not de-regulated in *Jarid2* ^*Δpanc*^ pancreases, suggesting that Jarid2 has broader effects and its primary function is not cell cycle gene regulation. Interestingly, during heart development, Jarid2 effects on the cell cycle are likely context-dependent differing temporarily and among the distinct cell layers of the heart^36^. In the skin, Jarid2 is required for the scheduled activation of adult stem cells in the hair follicle, through the inhibition *Cdkn2a* expression^16^. In our study, differentiated embryonic β-cells fail to re-entry the cell cycle at the end of gestation but we did not detect up-regulation of *Cdkn2a (p16/ink4a)* expression. Previous studies in mouse have shown that Jarid2 is required during heart formation and in neural cells within the brain to repress *CyclinD1* and promote differentiation^14, 25–28, 37^. Contrarily, in the pancreas, loss of Jarid2 results in the opposite effect. In this scenario and considering the preceding defects in endocrine cell differentiation, it is also possible that the consequences of Jarid2 loss in β-cell proliferation are secondary to their impaired differentiation. Supporting this notion, specific deletion of *Jarid2* in β-cells after the onset of insulin expression does not affect neonatal beta cell mass as assessed by morphometric analysis.

A previous study reported that *in vitro* generated β-cells display aberrant H3K27me3 and H3K4me3 modification patterns, indicating that the directed differentiation protocol failed to completely recapitulate the epigenetic changes that occur *in vivo* ^38^. In fact, it has been suggested that the generated α- and β-cells are missing important epigenetic modifications, and that histone modifying enzymes may be involved in fine-tuning development and function of pancreatic cell types^4–6^. Thus, in this context, our findings that Jarid2 regulates transcriptional priming of β-cell differentiation genes *in vivo* may have important implications in the development of stem cell-based and cell replacement therapies and therefore, the role of Jarid2 in the *in vitro* generation of β-cells warrants further investigation.

## Methods

### Mice

Mice were bred and maintained at the barrier animal facility of the University of Barcelona. Embryonic tissues were collected at indicated times, considering the morning of the appearance of a vaginal plug as embryonic day (E) 0.5. Principles of laboratory animal care were followed (European and local government guidelines) and animal procedures were approved by the Animal Research Committee of the University of Barcelona. Animals were euthanized by cervical dislocation. Pdx1-Cre^39, 40^, Neurog3-Cre^41^, Rip-Cre^42^, Jarid2^flox/flox^ ^43^, membrane-targeted tdTomato^44^ and R26R-EYFP^45^ mice have been described previously. Mice were genotyped with primers provided in Table S2. Animals with deletion of Jarid2 in early pancreatic progenitors, in endocrine progenitors and in β-cells crossing mice carrying a conditional allele of Jarid2 (*Jarid2* ^*flox*^) with transgenic Pdx1-Cre, Neurog3-Cre and Rip-Cre, respectively.

### Insulin content

Pancreases from newborn mice were dissected and proteins were recovered using a standard acid extraction and insulin content was quantified by ELISA assays (Mercodia, Uppsala, Sweden).

### Intraperitoneal Glucose Intolerance Test (ipGTT)

The ipGTT was performed after 5–6 h of food deprivation by administration of an injection of D-glucose (2 g/Kg body weight), and glycaemia from tail vein blood samples was measured at 0, 15, 30, 60 and 120 minutes after injection using a clinical glucometer and Accu-Check test strips (Roche Diagnostics, Switzerland).

### Immunofluorescence and morphometric analysis

Embryonic and P0 pancreases were fixed in 4% paraformaldehyde (PFA) (Electron Microscopy Sciences, Hatfield, PA) for 3–6 h. Adult pancreases were fixed in 4% formalin overnight. Tissues were subsequently washed, dehydrated, embedded in paraffin wax, and sectioned at 3 μm. For immunofluorescence, a standard immunodetection protocol was followed as described in ref. 46. Briefly, tissues were rehydrated and, when required, subject to heat-mediated antigen retrieval in citrate buffer. After a blocking step in 5% donkey serum/0.2% Triton X-100, tissue sections were incubated overnight with primary antibodies and then for 1 h with secondary antibodies (Table S3). Nuclei were stained with Hoechst 33258 (Sigma). Fluorescent images were captured using a Leica DMI 6000B widefield microscope or a Leica TCS SPE confocal microscope. For morphometrical analysis, e15.5 pancreas was sectioned at 3 μm and distributed as serial sections onto sets o or 5 slides. At least 10 sections 45 μm apart per animal for area, insulin and glucagon morphometries were analyzed. For adult pancreas morphometry, 6 sections per animal (2 × tail, 2 × body and 2 × head) were analyzed. For Ngn3+ morphometry, 4–6 sections per animal were analyzed. For ChgA morphometry, >2000 ChgA+ cells per animal were counted. For Ki67 and PHH3 morphometries, 2500–3000 insulin+ nuclei were counted. Morphometric analyses were performed using ImageJ software (http://rsb.info.nih.gov/ij/index.html).

### RNA isolation

Total RNA was isolated from tissues using the RNeasy kit (Qiagen, Hilden, Germany) and from sorted cells using the NucleoSpin XS RNA kit (Mackerey-Nagel, Düren, Germany). First-strand cDNA was prepared using the Superscript III RT kit and random hexamer primers (Invitrogen, Carlsbad, CA, USA). Reverse transcription reaction was carried for 90 min at 50 °C and an additional 10 min at 55 °C.

### Real-time PCR

Real time PCR (qRT-PCR) was performed on an ABI Prism 7900 sequence detection system using SybrGreen reagents: Express Greener (Invitrogen, Carlsbad, CA, USA) or GoTaq® qPCR Master Mix (Promega Biotech Ibérica, Alcobendas, Madrid, Spain). Expression relative to a housekeeping gene was calculated using the deltaCt method. We picked the moderately expressed gene *Tbp* as housekeeping for all the genes except for the pancreatic hormones and *Iapp*, which are more abundant, and whose expression was compared to that of *Actb*. Primer sequences are provided in Table S2.

### Global gene expression profiling

RNA from embryonic pancreases was hybridized onto GeneChip® Mouse Genome 430 2.0 Array (Affymetrix). Total RNA was isolated using the RNeasy kit (Qiagen, Hilden, Germany). Expression data were normalized with RMA, and the LIMMA package was used for statistical analysis to identify differentially expressed genes. Data have been deposited in Gene Expression Omnibus (www.ncbi.nlm.nih.gov/geo), accession number GSE77238. The DAVID Functional Annotation Tool (http://david.abcc.ncifcrf.gov) was used to identify enriched functional categories in differentially expressed genes (log_2_Fc < −0.29).

### Calculation of tissue-specificity scores

Publicly available gene expression profiles of pancreatic islets, β-cells, exocrine pancreas, liver, cerebral cortex, hypothalamus, skeletal muscle, kidney, lung and spleen^7, 22, 47^ were downloaded and normalized together with the embryonic pancreas samples. From these data, we generated a tissue-specificity score for each gene in each tissue, defined as the log2-ratio between the expression of a particular gene in the reference tissue and the median of its expression in all tissues, and considered a value > 4 to indicate tissue-specificity.

### Pancreatic cell dispersion and flow cytometry

Pancreatic buds were harvested from e15.5 embryos and treated with 0.125% trypsin-EDTA (Life Technologies) and 50ng/ml Dnase I (Stem cell) with agitation for 10–15 min at 37 °C. Digestion was inactivated by addition of 0.4volumes of FBS (falta provider). Transduced mPAC cells were collected after trypsinization in 15 ml tubes, pelleted and ressuspended to ∼2 × 10^6^ cells/ml in RPMI+3% FBS. Sorting was performed using a BD FACSAria SORP (for tomato) or BD FACSariaII (for GFP and YFP) machine, and cells were recovered in RNA lysis buffer or FBS for RNA extraction and ChIP assays, respectively.

### Cell culture and viral treatment

mPAC cells were grown in DMEM-4.5 g/L glucose (Sigma-Aldrich, St Louis, MO, USA) plus antibiotics supplemented with 10% FBS. For adenoviral transduction experiments, mPAC cells were seeded onto 10 cm plates and treated one day later with Ad.Ngn3-GFP adenoviruses (purified) at a multiplicity of infection (moi) of 10 unless otherwise indicated O/N. Cells were harvested for FACS sorting ∼42 h after transduction. The bicistronic adenovirus encoding Ngn3-GFP was kindly provided by Dr. H. Heimberg^48^.

### Chromatin immunoprecipitation

E15.5 embryonic pancreases were fixed in 1% formaldehyde for 10 min at room temperature with rotation. Fixation was quenched with 0.125 M glycine for 5 min, and pancreases were washed three times for 5–10 min in PBS and stored at −80 °C until further processing. Histone ChIPs were performed as described elsewhere^49^. ChIPs against RNAPII-Ser5P were performed following the protocol described by Stock JK, Bookes E and Pombo A in http://www.epigenesys.edu/en/protocols (prot48) with some modifications: 3–6 e15.5 fixed pancreases were resuspended in 150–200 μl SDS Lysis Buffer supplemented with phosphatase and protease inhibitors and sonicated in a Bioruptor (Diagenode) for 6 cycles of 5 min each (30 sec ON 30 sec OFF) at High setting. After removing cellular debris by centrifugation chromatin was diluted 10-fold in ChIP dilution buffer and 30–70 μg were immunoprecipitated with 3 μg of Anti-RNA polymerase II CTD repeat YSPTSPS antibody [H14] - ChIP Grade (Abcam) and 20 μl of bridged ProteinG Dynabeads (Life Technologies) as described by Stock JK, Bookes E and Pombo A in http://www.epigenesys.eu/en/protocols (prot48). Washes were performed as described by Stock JK *et al*. and elutions as described in ref. 49. For histone ChIPs from sorted pancreatic cells, we adapted the protocol for low-cell number ChIP detailed in ref. 9. Briefly, 30,000–60,000 cells were collected in 50 ul FBS, and fixed in 500 ul with 1% formaldehyde for 10 min at room temperature with rotation. Fixation was quenched with 0.125 M glycine for 5 min and cells were washed as described by Xu *et al*.^9^. Cells were resuspended in 100 ul of SDS-lysis buffer and stored at −80 °C until further processing. After storage, *Jarid2* ^wt^ and *Jarid2* ^Δendo^ cells were processed in parallel. Chromatin was diluted 10-fold in ChIP dilution buffer before immunoprecipitation with 1 μg of the antibodies listed in Table S3. Immunoprecipitated chromatin (specific antibody and IgG control) was analyzed, together with inputs, by real-time PCR as described above. Percentage of input was calculated as follows:

$${{\rm{2}}}^{-({\rm{Ct\; antibody}} \mbox{-} {\rm{Ct\; input}})}-{2}^{-({\rm{Ct\; IgG}} \mbox{-} {\rm{Ct\; input}})}.$$

### Immunoblotting

Embryonic pancreases were lysed in triple detergent lysis buffer (Tris-HCl 50 mM, NaCl 150 mM, 0.1% SDS, 1% NP40 and 0.5% Sodium Deoxycholate) and extraction of histones was optimized through the addition of HCl to a final concentration of 0.2 N. After 30 min incubation on ice, cell debris was pelleted and supernatants were quantified and prepared for SDS-PAGE electrophoresis on 16% Tris-tricine homemade gels. Proteins were then transferred to a Polyscreen PVDF membrane (Perkin Elmer, Waltham, MA, USA) and incubated overnight at 4 °C with the antibodies indicated Table S3. Blots were visualized with ECL Reagent (Pierce Biotechnology, Rockford, IL, USA) using a LAS4000 Lumi-Imager (Fuji Photo Film, Valhalla, NY). Protein spots were quantitated with Image J software (http://rsb.info.nih.gov/ij/index.html).

### Statistics

Data are presented as mean ± standard error of the mean (SEM). Statistical significance was tested using unpaired Student’s t-test, or 2-way ANOVA for the ipGTT.

### Study approval

Animal procedures were approved by the Animal Research Committee of the University of Barcelona.

## Electronic supplementary material

Supplemental Data
