# Supplementary material for: Late-stage differentiation of embryonic pancreatic β-cells requires Jarid2
Source: Sci Rep. 2017 Sep 14;7:11643. doi: 10.1038/s41598-017-11691-2 (PMC5599523; doi:10.1038/s41598-017-11691-2)
Supplement: Supplementary file 1 — Supplemental Data [file 41598_2017_11691_MOESM1_ESM.pdf]

Late-stage differentiation of embryonic pancreatic β-cells requires Jarid2

Sara Cervantes[1,2,*], Marta Fontcuberta-Pi Sunyer[1,2,3], Joan-Marc Servitja[1,2], Rebeca Fernandez-Ruiz[1,2], Ainhoa García[1,2], Lidia Sanchez[1,2], Young-Sook Lee[4], Ramon Gomis[1,2,5,6], Rosa Gasa[1,2,*]

[1] Diabetes and Obesity Research Laboratory, August Pi i Sunyer Biomedical Research Institute (IDIBAPS), Rosselló 149-153, 08036 Barcelona, Spain

[2] Centro de Investigación Biomédica en Red de Diabetes y Enfermedades Metabólicas Asociadas (CIBERDEM), Spain

[3] University of Barcelona, Barcelona, Spain

[4] Department of Cellular and Regenerative Biology, University of Wisconsin-Madison, Madison, Wisconsin 53706, USA.

[5] Department of Endocrinology and Nutrition, Hospital Clinic of Barcelona, Barcelona, Spain

[6] Department of Medicine, University of Barcelona, Barcelona, Spain

[*] Corresponding authors

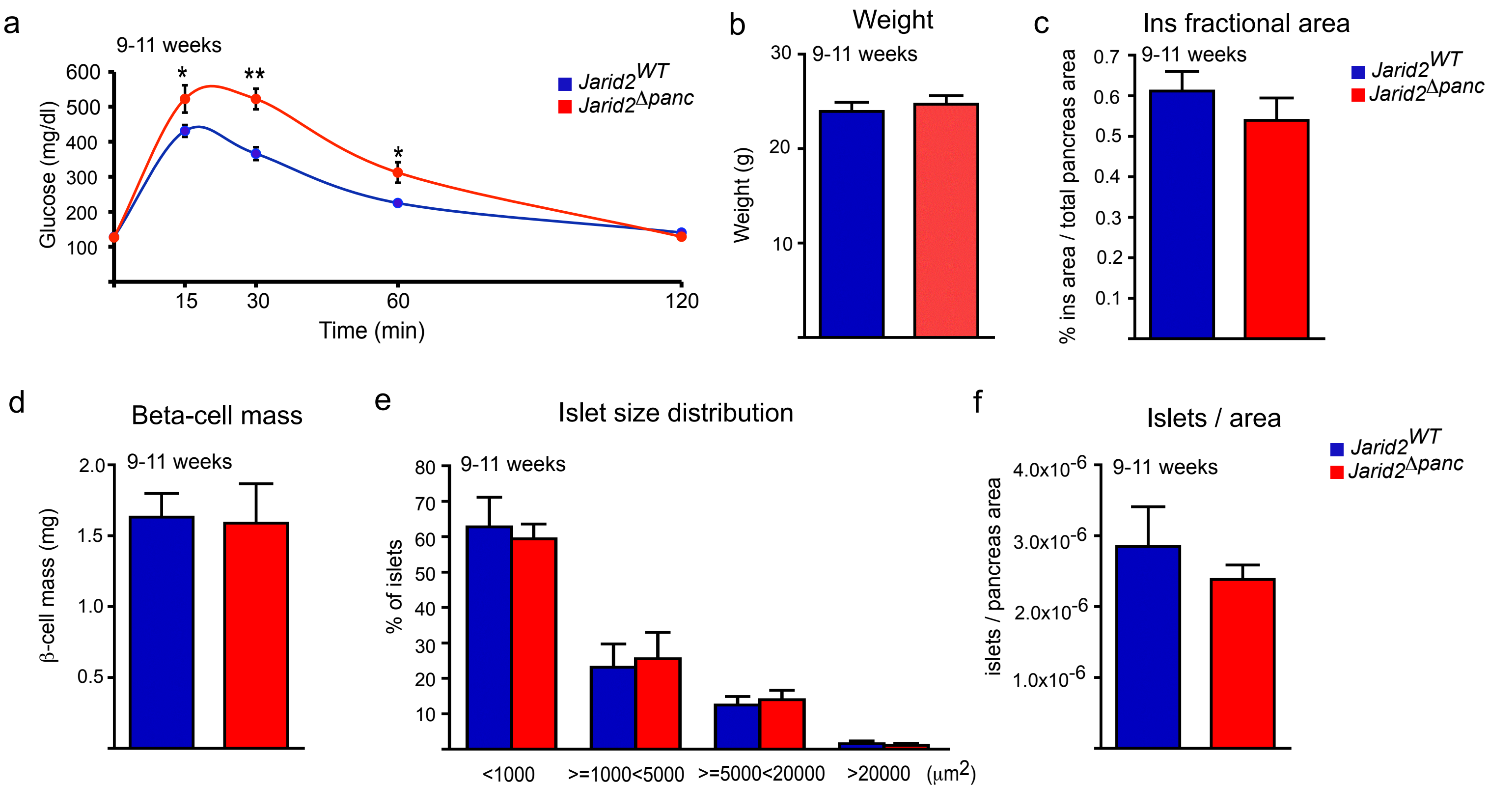

Figure S1. Ablation of Jarid2 in pancreatic progenitors results in glucose intolerance in adult mice. (a) Intraperitoneal Glucose Tolerance Test (ipGTT) in 9-11 week-old *Jarid2*^{WT} (n=8) and *Jarid2*$^{\Delta panc}$ (n=9) male mice were performed after 5-6 hrs of fasting. Each datapoint represents mean ± SEM. *P<0.05, **P<0.001. **(b)** Body weight of 9-11 week-old *Jarid2*^{WT} (n=8) and *Jarid2*$^{\Delta panc}$ (n=9) male mice after 5-6 hrs of fasting. Bars represent mean ± SEM. **(c-f)** Pancreas from 12 week-old *Jarid2*^{WT} (n=3) and *Jarid2*$^{\Delta panc}$ (n=3) were harvested, fixed, sectioned and immuno-stained for insulin: **(c)** Fractional insulin-positive area (relative to total pancreatic area); **(d)** Beta cell mass (fractional insulin-positive area x pancreas weight); **(e)** Islet size distribution, expressed as percentage of total islets; **(f)** Number of islets per pancreatic area. Bars represent mean ± SEM. Similar results were obtained with pancreas from adult females (data not shown).

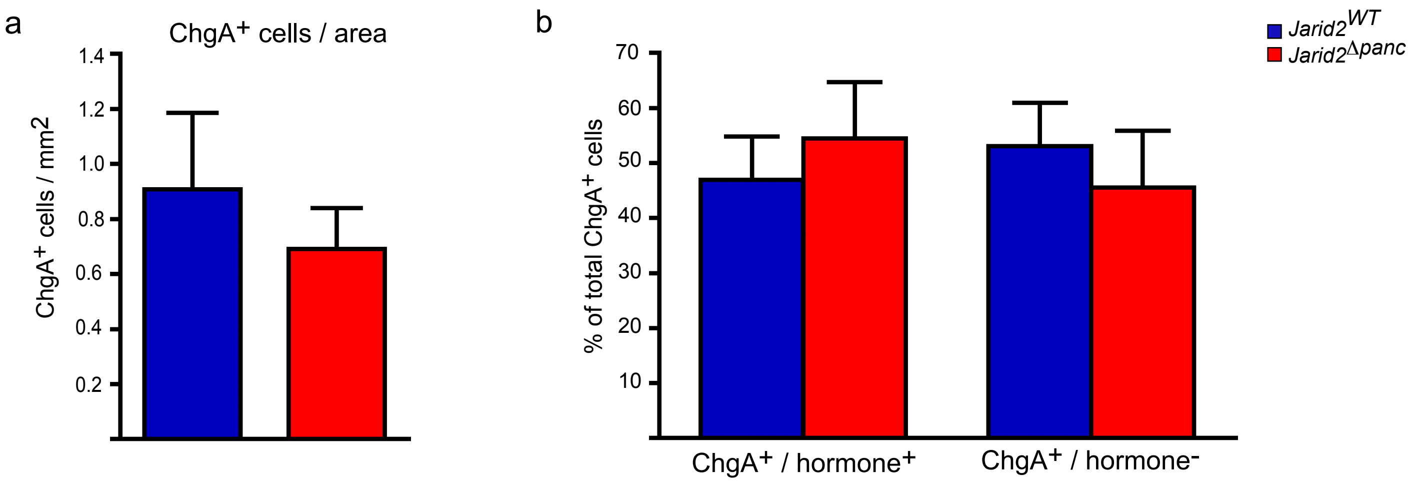

Figure S2. Ablation of Jarid2 in pancreatic progenitors does not impair endocrine cell commitment. Pancreas from e15.5 *Jarid2*^{WT} (n=3) and *Jarid2*$^{\Delta panc}$ (n=3) embryos were harvested, fixed, sectioned and immunostained for Chromogranin A (ChgA), Pdx1, insulin and glucagon. (a) Number of ChgA+ cells per Pdx1+ (epithelial) area analyzed. (b) Percentage of total number of ChgA+ cells that were hormone+ (insulin and glucagon) or hormone-. Note that there were no differences in either population between *Jarid2*$^{\Delta panc}$ and *Jarid2*^{WT}. Bars represent mean ± SEM

a

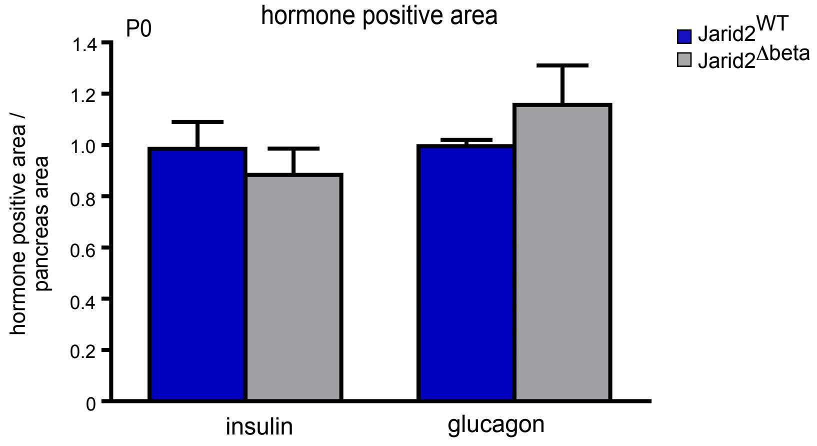

Figure S3. Ablation of Jarid2 in beta cells does not result in defects in insulin or glucagon positive cell area in newborn mice. Morphometric analysis of *Jarid2*^{WT} (n=3) and *Jarid2*$^{\Delta beta}$ (n=3) insulin and glucagon positive area in newborn mice (P0). Bars represent mean ± SEM.

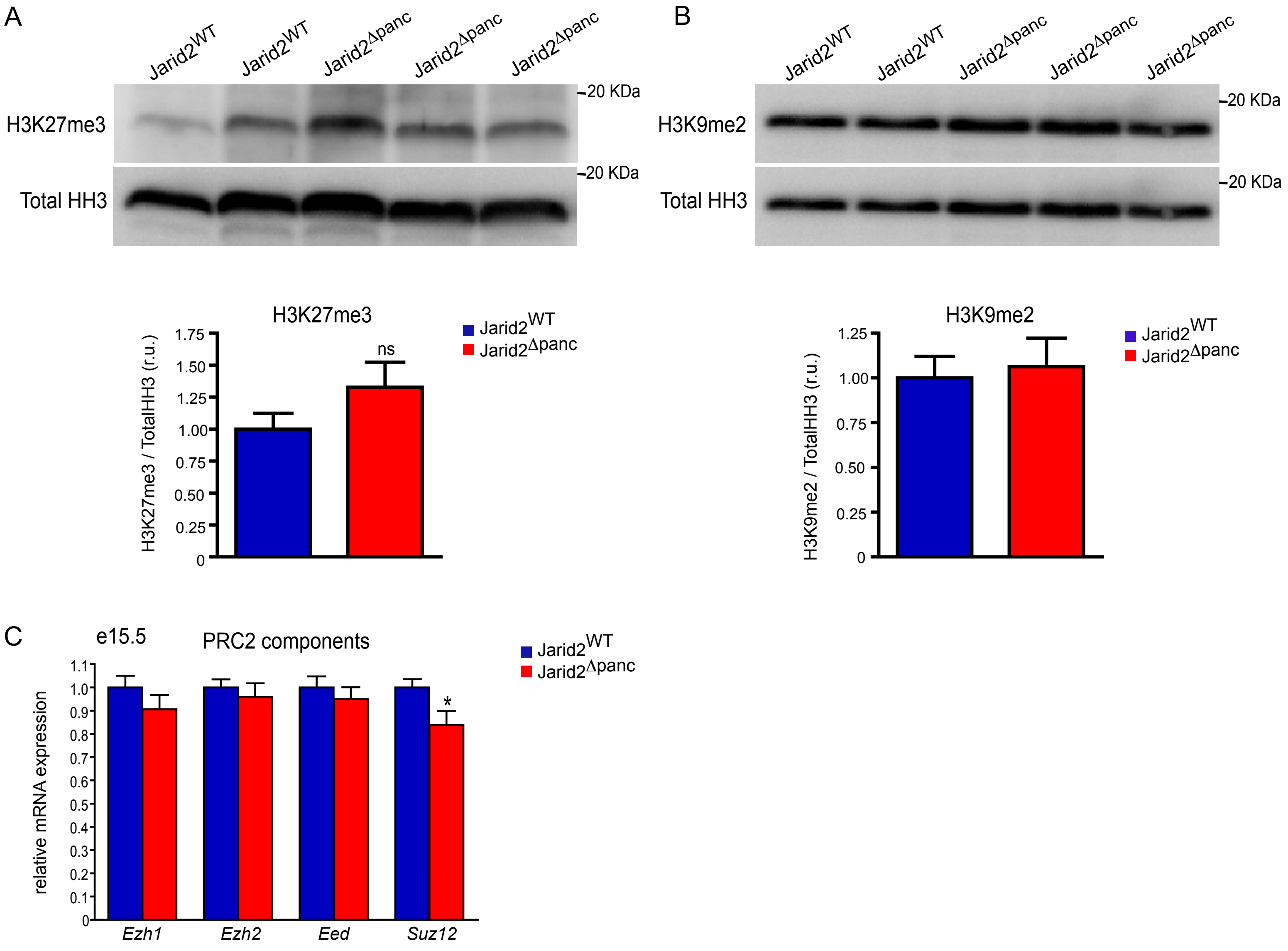

Figure S4. Lack of major changes in H3K27me3 and H3K9me2 in the absence of Jarid2
(a) Quantification by Western blot of H3K27me3 levels in total pancreatic lysates from individual e15.5 *Jarid2^{WT}* (n=10) and *Jarid2$^{\Delta panc}$* (n=8) embryonic pancreas. A representative image is shown. Total Histone H3 was used as loading control. Bars represent mean ± SEM. (b) Quantification by Western blot of H3K9me2 levels in total pancreatic lysates from individual e15.5 *Jarid2^{WT}* (n=4) and *Jarid2$^{\Delta panc}$* (n=3) embryonic pancreas. A representative image is shown. Total Histone H3 was used as loading control. Bars represent mean ± SEM. (c) Quantification by qRT-PCR of the relative expression of the PRC2 core components *Ezh1*, *Ezh2*, *Eed* and *Suz12* at e15.5 in *Jarid2^{WT}* (n=11) and *Jarid2$^{\Delta panc}$* (n=15) embryonic pancreases. Bars represent mean ± SEM. *P<0.05

Table S1. Endocrine gene expression.
Comparison of expression of selected endocrine genes in pancreases from *Jarid2*$^{\Delta panc}$ and control embryos at E15.5.

ND= Not determined.

Probe Set ID Best p-value Jarid2	Gene Title	Gene Symbol	Significantly changed by qPCR	logFC	P.Value	adj.P.Val	logFC BEST Ngn3-YFP	P.Value	adj.P.Val
1421112_at	NK2 transcription factor related, locus 2 (Drosophila)	**Nkx2-2**	NO	0,0	0,79	0,96	4,38	2,3E-08	1,6E-05
1457613_at	---	**Rfx6**	NO	-0,1	0,63	0,92	4,36	8,8E-09	1,0E-05
1425828_at	NK6 homeobox 1	**Nkx6-1**	NO	0,1	0,60	0,91	3,45	1,2E-05	5,9E-04
1450042_at	aristaless related homeobox	**Arx**	ND	-0,1	0,44	0,86	5,62	7,5E-10	2,7E-06
1450723_at	ISL1 transcription factor, LIM/homeodomain	**Isl1**	ND	-0,2	0,14	0,71	3,55	2,3E-07	6,3E-05
1422773_at	myelin transcription factor 1	**Myt1**	ND	-0,2	0,14	0,70	4,42	2,1E-08	1,6E-05
1430353_at	GLIS family zinc finger 3	**Glis3**	ND	-0,1	0,10	0,65	2,36	5,0E-06	3,5E-04
1451598_at	paired box gene 4	**Pax4**	NO	-0,2	0,09	0,64	4,24	7,8E-10	2,7E-06
1415885_at	chromogranin B	**Chgb**	NO	-0,2	0,08	0,63	5,19	3,9E-07	7,9E-05
1418149_at	chromogranin A	**Chga**	NO	-0,2	0,06	0,60	5,50	1,9E-09	4,4E-06
1422312_a_at	neurogenin 3	**Neurog3**	NO	-0,2	0,02	0,50	3,15	1,9E-07	5,5E-05
1448676_at	calcium/calmodulin-dependent protein kinase II, beta	**Camk2b**	ND	-0,2	0,05	0,58	2,39	4,1E-05	1,3E-03
1419271_at	paired box gene 6	**Pax6**	YES	-0,3	0,05	0,58	6,17	1,5E-08	1,4E-05
1455865_at	insulinoma-associated 1	**Insm1**	YES	-0,2	0,03	0,51	5,45	7,7E-09	1,0E-05
1420440_at	pancreatic polypeptide	**Ppy**	ND	-0,3	0,02	0,47	4,99	5,8E-08	2,7E-05
1426412_at	neurogenic differentiation 1	**Neurod1**	YES	-0,4	0,01	0,41	5,78	2,6E-10	2,3E-06
1425952_a_at	glucagon	**Gcg**	YES	-0,5	0,00	0,32	3,73	3,6E-06	2,9E-04
1448980_at	ghrelin	**Ghrl**	ND	-0,4	0,00	0,25	4,31	4,2E-06	3,2E-04
1421396_at	proprotein convertase subtilisin/kexin type 1	**Pcsk1**	YES	-0,6	0,00	0,24	5,58	1,2E-07	4,4E-05
1423529_at	glucose-6-phosphatase, catalytic, 2	**G6pc2**	YES	-1,1	0,00	0,21	5,37	1,7E-10	2,3E-06
1451716_at	v-maf musculoaponeurotic fibrosarcoma oncogene family, protein B (avian)	**Mafb**	YES	-0,5	0,00	0,20	4,50	1,0E-08	1,1E-05
1448628_at	secretogranin III	**Scg3**	ND	-0,7	0,00	0,14	5,55	6,2E-08	2,8E-05
1436092_at	---	**Mafa**	YES	-1,0	0,00	0,14	5,98	5,6E-09	9,6E-06
1460081_at	synaptotagmin VII	**Syt7**	ND	-0,5	0,00	0,14	4,99	4,7E-08	2,4E-05
1423506_a_at	neuronatin	**Nnat**	ND	-0,4	0,00	0,13	3,53	4,6E-06	3,4E-04

1417954_at	somatostatin	**Sst**	YES	-0,6	0,00	0,12	5,44	8,4E-07	1,2E-04
1444027_at	solute carrier family 30 (zinc transporter), member 8	**Slc30a8**	ND	-1,2	0,00	0,11	5,18	4,1E-08	2,2E-05
1435064_a_at	transmembrane protein 27	**Tmem27**	ND	-0,5	0,00	0,11	5,26	2,6E-08	1,6E-05
1450708_at	secretogranin II	**Scg2**	ND	-0,8	0,00	0,09	5,83	2,3E-08	1,6E-05
1422447_at	insulin I	**Ins1**	YES	-0,9	0,00	0,08	4,47	1,6E-08	1,5E-05
1448312_at	proprotein convertase subtilisin/kexin type 2	**Pcsk2**	ND	-0,6	0,00	0,04	5,87	5,4E-11	2,3E-06
1422446_x_at	insulin II	**Ins2**	YES	-0,9	0,00	0,03	3,86	9,4E-06	5,0E-04
1419127_at	neuropeptide Y	**Npy**	YES	-1,2	0,00	0,03	4,35	5,4E-07	9,4E-05
1417988_at	regulated endocrine-specific protein 18	**Resp18**	ND	-0,8	0,00	0,02	4,57	9,8E-08	3,7E-05
1437323_a_at	islet amyloid polypeptide	**Iapp**	YES	-1,1	0,00	0,02	5,24	9,0E-09	1,0E-05
1426225_at	retinol biNDing protein 4, plasma	**Rbp4**	ND	0,0	0,79	0,96	4,71	1,1E-07	4,2E-05
1449067_at	solute carrier family 2 (facilitated glucose transporter), member 2	**Slc2a2**	YES	-0,1	0,44	0,85	5,26	7,0E-09	1,0E-05
1422330_at	glucagon-like peptide 1 receptor	**Glp1r**	ND	-0,1	0,33	0,81	0,15	4,9E-01	7,7E-01
1419146_a_at	glucokinase	**Gck**	YES	0,2	0,15	0,71	3,47	1,4E-09	4,2E-06

Table S2. List of oligonucleotides

GENE	SEQUENCE	APLICATION
Actb	5’ TGAGAGGGAAATCGTGCGTG<br>3’ TGCTTGCTGATCCACATCTGC	RT-PCR
Actb prox	5’ AACAAGAGGCCACACAAATAGG<br>3’ ACCCTCTGGGTGTGGATGTC	ChIP-qPCR
Amy	5’ TGGCGTCAAATCAGGAACATG<br>3’ AAAGTGGCTGACAAAGCCCAG	qRT-PCR
Ccna2	5’ CGACGGGTTGCTCCTCTTA<br>3’ CTGAGTCTCTTCTGCTTCATCC	qRT-PCR
Ccnd1	5’ GCGTACCCTGACACCAATCTC<br>3’ CTCCTCTTCGCACTTCTGCTC	qRT-PCR
Ccnd2	5’ GCCAAGATCACCCACACTGATG<br>3’ TCCGGCGTTATGCTGCTCT	qRT-PCR
Ccnd3	5’ CGAGCCTCCTACTTCCAGTG<br>3’ GGACAGGTAGCGATCCAGGT	qRT-PCR
Cdkn1a	5’ CCTGGTGATGTCCGACCTG<br>3’ CCATGAGCGCATCGCAATC	qRT-PCR
Cdkn1b	5’ ATGAGGAAGCGACCTGCT<br>3’ CCACAGTGCCAGCGTTC	qRT-PCR
Cdkn1c	5’ AGCAGGACGAGAATCAAGAG<br>3’ GAAGAAGTCGTTCGCATTGG	qRT-PCR
Cdk4	5’ ATCAAGGTCACCCTAGTGTTTG<br>3’ AGAAACTGACGCATTAGATCCTTA	qRT-PCR
Cdk6	5’ AGTGCAGACCAGTGAGGA<br>3’ CAACCTGACCACGTTGGG	qRT-PCR
Chga	5’ AGGGGACACCAAGGTGATGA<br>3’ AGCAGATTCTGGTGTCGCAG	qRT-PCR
Chgb	5’ GAATTGGGGATATGAGAAGAGAAGC<br>3’ AGATCCATCGCAGCCAAGTTC	qRT-PCR
Cre	5’ TGCCACGACCAAGTGACAGC<br>3’ CCAGGTTACGGATATAGTTCATG	genotyping
Eed	5’ GCGATGGTTAGGCGATTTGAT<br>3’ TTTTGCCAGGTTTCCAGCAT	qRT-PCR
Ezh1	5’ GGGCCTCCAGTTCTTCAGAGGCT<br>3’ TCCGGTCCATTCCACCGGCT	qRT-PCR
Ezh2	5’ GCGGGACTAGGGAGTGTTCA<br>3’ AGGGTCTTTAACGGGATGACTTG	qRT-PCR
FoxM1	5’ TGAGGGTCAAAGCTTGCGAT<br>3’ TCTGATGTTTCACTCGGGGC	qRT-PCR
G6pc2	5’ CAGGAGGACTACCGGACTTAC<br>3’ TCAACTGAAACCAAAGTGGGAA	qRT-PCR<br>ChIP-qPCR
Gcg	5’ AGGAATTCATTGCGTGGCTG<br>3’ CAATGGCGACTTCTTCTGGG	qRT-PCR
Iapp	5’ CTCCAAACTGGCAGGTGTCC<br>3’ TCCGTTTGTCCATCTGAGGG	qRT-PCR
IL-2	5’ CCAGGTTACGGATATAGTTCATG<br>3’ GTA GGT GGA AAT TCT AGC ATC ATC C	genotyping (control)
Ins1 prox	5’ TGACCAATGAGTGGGCTACG<br>3’ ATGAGATCCCAGCTCACCCT	ChIP-qPCR
Ins1+2	5’ AGCGTGGCTTCTTCTACACACC<br>3’ CCAGCTCCAGTTGTGCCACT	qRT-PCR
Insm1 prox	5’ CTTTATTCCGCAGCGCCTTG<br>3’ GAGCGGGCATTTTGTCTGTG	ChIP-qPCR
Insm1	5’ CTGGCGGCGTATCCGAATC<br>3’ CCTGGCGACGGAACTTCTT	qRT-PCR
Jarid2 exon3	5’ AGGCCTTGCCGGGAGCCTGAA<br>3’ AGCAAAGGCTCCACTATCTTC	qRT-PCR
Jarid2 3’	5’ GCACTTGTGCTACCTGTCCA<br>3’ GCCAGACACTTTGCCACATA	qRT-PCR
Jarid2 flox	5’ GCGGTAAATGGTGAGTTGAAA<br>3’ ACAGACTGACACACCTTCC	genotyping
Ki67	5’ CAGCTCCTGCCTGTTTGGAA<br>3’ TTGCCTCTTGCTCTTTGACTTCA	qRT-PCR

MafA	5’ CAAGGAGGAGGTCATCCGAC<br>3’ TCTCCAGAATGTGCCGCTG	qRT-PCR
MafA prox	5’ GTCACCGGTGTCAGGATAGT<br>3’ GGGTACTCCTTCGGTGTCTC	ChIP-qPCR
MafB	5’ TGGATGGCGAGCAACTACC<br>3’ CCAGGTCATCGTGAGTCACA	qRT-PCR
MyoD prox	5’ CATTGTCCCGTAGCCTTGAG<br>3’ GCCACACGCGGTAGCACTTG	ChIP-qPCR
NeuroD1	5’ GGATCAATCTTCTCTTCCGGTG<br>3’ TGCGAATGGCTATCGAAAGAC	qRT-PCR
NeuroD1 prox	5’ CCACAAAGGGTTAATCTCTCC<br>3’ CGTCCAGACTGAACGACTCC	ChIP-qPCR
Neurog3 (mouse)	5’ TTCTCATCGGTACCCTTGCTG<br>3’ GCAGACTCACCAGGAAGTATGG	qRT-PCR
Nkx2-2	5’ GCCTCCAATACTCCCTGCAC<br>3’ GTCATTGTCCGGTGACTCGT	qRT-PCR
Nkx6-1	5’ TGGACAGCAAATCTTCGCCCTG<br>3’ TGTTGTAATCGTCGTCATCCTC	qRT-PCR
Pak3	5’ CCATGTGCACACCTCTGACT<br>3’ TCACCTGATGGCAGCTTCTG	qRT-PCR
Pax4	5’ GAGTACCCTGCTCTTTTTGCC<br>3’ ACTCGATTGATAGAGGACACACT	qRT-PCR
Pax6	5’ TACCAGTGTCTACCAGCCAAT<br>3’ TGCACGAGTATGAGGAGGTCT	qRT-PCR
PCNA	5’ ATCGTGAATCGGGGG<br>3’ AAACATGGTGGCGGA	qRT-PCR
Pcsk1 prox	5’ TTAATGAAACTTGCCACCTCCC<br>3’ ACCCCAGATCATTTGGAGCCC	ChIP-qPCR
Pcsk1	5’ TTAATGAAACTTGCCACCTCCC<br>3’ ACCCCAGATCATTTGGAGCCC	qRT-PCR
Pdx1	5’ CCCCAGTTTACAAGCTCGCT<br>3’ CTCGGTTCCATTCGGGAAAGG	qRT-PCR
Rfx6	5’ CCTTAGTAATGCAGGAGCTGGC<br>3’ TCCAAGCCTATGTGCCTCTGA	qRT-PCR
Ripply3	5’ TGAGTCTTGGGGAGACCAAC<br>3’ AGAAATGAATGGTGGCTTGC	qRT-PCR
R26-YFP	5’ AAAGTCGCTCTGAGTTGTTAT<br>3’ GCGAAGAGTTTGTCCTCAACC<br>GGAGCGGGAGAAATGGATATG	genotyping
Sst	5’ ACCCCAGACTCCGTCAGTTTC<br>3’ ATCATTCTCTGTCTGGTTGGGC	qRT-PCR
Sst prox	5’ GATGCCTCTGCTCTTCCAACT<br>3’ CACAGGAGGGAGGGGGAGAA	ChIP-qPCR
Suz12	5’ GAAGCTGTGGAACCTCCATGTC<br>3’ ACAGCATACAGGCATGATTCATTT	qRT-PCR
Tbp	5’ ACCCTTCACCAATGACTCCTATG<br>3’ ATGATGACTGCAGCAAATCGC	qRT-PCR
tdTomato	5’ WT AAGGGAGCTGCAGTGGAGTA<br>3’ WT CCGAAAATCTGTGGGAAGTC<br>5’ mut CTGTTCCTGTACGGCATGG<br>3’ mut GGCATTAAAGCAGCGTATCC	genotyping

Table S3. List of antibodies

WB: western blot; IF: immunofluorescence; ChIP

PRIMARY ANTIBODIES	RAISED IN	DILUTION	SOURCE
amylase	rabbit	1:200 (IF)	Sigma-Aldrich, St Louis, MO, USA
chromograninA	rabbit	1:200 (IF)	Thermo Scientific
Foxa2	goat	1:100	Santa Cruz Biotechnologies
GFP	chicken	1:200 (IF)	Abcam, Cambridge, UK
glucagon	mouse	1:500-1:1000 (IF)	Sigma-Aldrich
insulin	guinea pig	1:500-1:1000 (IF)	DAKO, Glostrup, Denmark
insulin	rabbit	1:500-1:1000 (IF)	Santa Cruz Biotechnologies
Ki67	rabbit	1: 200 (IF)	Thermo Scientific
Muc-1	rabbit	1:200 (IF)	Santa Cruz Biotechnologies
neurogenin3	mouse	1:2000 (IF)	Developmental Studies Hybridoma Bank, Iowa City, IA, USA
Nkx2-2	mouse	1: 500 (IF)	Developmental Studies Hybridoma Bank, Iowa City, IA, USA
Nkx6-1	mouse	1:500 (IF)	Developmental Studies Hybridoma Bank, Iowa City, IA, USA
Pdx1	guinea pig	1:500 (IF)	Abcam, Cambridge, UK
PHH3	rabbit	1:200 (IF)	Millipore, Billerica, MA, USA
somatostatin	rabbit	1:500 (IF)	DAKO, Glostrup, Denmark
Sox9	rabbit	1:2000 (IF)	Millipore, Billerica, MA, USA
Histone H3	rabbit	1:1000 (WB)	Abcam, Cambridge, UK
H3K9me2	mouse	1 ug/ ChIP reaction<br>1:1000 (WB)	Abcam, Cambridge, UK
H3K27me3	rabbit	1 ug/ ChIP reaction	Millipore, Billerica, MA, USA
H3K27me3	mouse	1:500 (WB)	Abcam, Cambridge, UK
RNAPII-Ser5p	Mouse IgM	3ug/ ChIP reaction	Abcam, Cambridge, UK

SECONDARY ANTIBODIES	RAISED IN	DILUTION	SOURCE
Anti-mouseIgM	rabbit	4 ug / 20ul dynabeads	Jackson ImmunoResearch, Suffolk, UK
Anti-mouse IgG peroxidase	sheep	1/5000	GE Healthcare
Anti-rabbit IgG peroxidase	donkey	1/5000	GE Healthcare
Cy2 anti-guinea pig	donkey	1/500	Jackson ImmunoResearch, Suffolk, UK
Alexa Fluor® 488 anti-guinea pig	donkey	1/500	Jackson ImmunoResearch, Suffolk, UK
Alexa Fluor® 555 anti-guinea pig	donkey	1/250	Molecular Probes
Cy3 anti-guinea pig	donkey	1/500	Jackson ImmunoResearch, Suffolk, UK
Alexa Fluor® 555 anti-mouse	donkey	1/250	Molecular Probes
Cy3 anti-mouse	donkey	1/500	Jackson ImmunoResearch, Suffolk, UK
Alexa Fluor® 488 anti-rabbit	donkey	1/500	Jackson ImmunoResearch, Suffolk, UK
Cy3 anti-rabbit	donkey	1/500	Jackson ImmunoResearch, Suffolk, UK
Alexa Fluor® 555 anti-rabbit	mouse	1/250	Molecular Probes
